# Identification and Pleiotropic Effect Analysis of *GSE5* on Rice Chalkiness and Grain Shape

**DOI:** 10.3389/fpls.2021.814928

**Published:** 2022-01-20

**Authors:** Liangrong Jiang, Hui Zhong, Xianbin Jiang, Jiaoping Zhang, Rongyu Huang, Furong Liao, Yaqin Deng, Qingqing Liu, Yumin Huang, Houcong Wang, Yi Tao, Jingsheng Zheng

**Affiliations:** ^1^Xiamen Plant Genetics Key Laboratory, School of Life Sciences, Xiamen University, Xiamen, China; ^2^Guangxi Rice Genetics and Breeding Key Laboratory, Rice Research Institute, Guangxi Academy of Agricultural Sciences, Nanning, China; ^3^National Key Laboratory for Crop Genetics and Germplasm Enhancement, Nanjing Agricultural University, Nanjing, China; ^4^Xiamen Entry-Exit Inspection and Quarantine Bureau, Xiamen, China

**Keywords:** rice (*Oryza sativa* L.), percentage of grains with chalkiness, degree of endosperm chalkiness, grain shape, near-isogenic lines, molecular marker-assisted selection, transcriptomics, proteomics

## Abstract

Chalkiness is one of several major restricting factors for the improvement of rice quality. Although many chalkiness-related quantitative trait loci have been mapped, only a small number of genes have been cloned to date. In this study, the candidate gene *GSE5* of a major quantitative trait locus (QTL) for rice chalkiness, *qDEC5*, was identified by map-based cloning. Phenotyping and haplotype analysis of *proActin:GSE5* transgenic line, *gse5-cr* mutant, and 69 rice varieties further confirmed that *GSE5* had the pleiotropic effects and regulated both chalkiness and grain shape. Genetic analysis showed *GSE5* was a dominant gene for grain length and a semi-dominant gene for grain width and chalkiness. The DNA interval closely linked to *GSE5* was introgressed to Zhenshan 97B (ZB) based on molecular marker-assisted selection, and the improved ZB showed lower chalkiness and longer but smaller grains, which showed that *GSE5* played an important role in breeding rice varieties with high yield and good quality. Transcriptomics, proteomics, and qRT-PCR analyses showed that thirty-nine genes associated with carbon and protein metabolism are regulated by *GSE5* to affect the formation of chalkiness, including some newly discovered genes, such as *OsCESA9*, *OsHSP70*, *OsTPS8*, *OsPFK04*, *OsSTA1*, *OsERdj3A*, etc. The low-chalkiness lines showed higher amino sugar and nucleotide sugar metabolism at 10 days after pollination (DAP), lower carbohydrate metabolism at 15 DAP, and lower protein metabolism at 10 and 15 DAP. With heat shock at 34/30°C, rice chalkiness increased significantly; *OsDjC10* and *OsSUS3* were upregulated at 6 and 12 DAP, respectively, and *OsGSTL2* was downregulated at 12 DAP. Our results identified the function and pleiotropic effects of *qDEC5* dissected its genetic characteristics and the expression profiles of the genes affecting the chalkiness formation, and provided a theoretical basis and application value to harmoniously pursue high yield and good quality in rice production.

## Introduction

Rice is a staple food for more than half of the global population. Previous studies showed that the current rice quality was generally inadequate and that the main factor that restricted improvement of rice quality was the chalkiness trait (Qi, 2011; Xu et al., 2011; Meng et al., 2012). The chalky part of the rice grain not only renders an unattractive appearance but also markedly affects the processing and cooking quality as well as the taste of rice grains (Qi, 2011). Hence, improving rice chalkiness has great significance for rice quality improvement.

Rice chalkiness is strongly influenced by environmental conditions, especially the temperature at the grain filling stage (Kentaro et al., 2016; Chen et al., 2017; Kabir et al., 2017; Cheng et al., 2019). However, great variations exist in the chalkiness trait among rice varieties. For example, the percentage of grains with chalkiness (PGWC) and the degree of endosperm chalkiness (DEC) of an elite rice variety, Jiafuzhan, is close to zero under various environmental conditions (Wang et al., 2007). Therefore, the chalkiness trait is mainly controlled by genetic factors, and some rice germplasms can maintain a low level of chalkiness regardless of different ecological regions (Cheng et al., 2019; Zhou et al., 2019). As a quantitative trait, chalkiness is polygenic and has a strong additive effect on inheritance characteristics (Jin et al., 2000). The primary task in decreasing rice chalkiness is to improve the chalkiness-related genetic characteristics. Hence, the major genes related to chalkiness must be identified and cloned to perform functional analyses and facilitate comprehensive dissection of the genetic network for the chalkiness trait.

To date, many researchers have conducted quantitative trait loci (QTLs) mapping and epistatic interaction analysis for rice chalkiness using different types of mapping populations and have altogether identified more than 400 QTLs (He et al., 1999; Tan et al., 2000; Zeng et al., 2002; Li et al., 2003, 2004, 2016; Wan et al., 2005; Liu et al., 2007; Zhou et al., 2009b,c; Gao et al., 2014, 2016; Qiu et al., 2015, 2017; Tao et al., 2015; Wang et al., 2016; Yun et al., 2016; Gong et al., 2017; Jiang et al., 2017; Misra et al., 2021). These QTLs can be further grouped into approximately 30 intervals by a comparative analysis and integration with high-density molecular marker linkage maps. These mapping results implied that some genetic loci would be present in different genetic mapping populations and under different environmental conditions. Cloning these genes and dissecting their functions are important for understanding the genetic network of rice chalkiness. To date, three major QTLs associated with rice chalkiness have been fine mapped (*qPGWC-7*, *QTLqPGWC-8*, and *qACE9*), which map to a 44-kb interval on chromosome 7 (Zhou et al., 2009a), a 142-kb interval on chromosome 8 (Guo et al., 2011) and a 22-kb interval on chromosome 9 (Li et al., 2014), respectively. *PGC6.1* was found to be a QTL contributing to lower grain chalkiness in low amylose varieties in a genome-wide association study (GWAS) (Misra et al., 2021).

With the development of “omics” analysis technology, some new technologies, such as transcriptomics, proteomics, and metabolomics, have been widely used to explore the complicated mechanism for the formation of rice chalkiness. The results showed that chalkiness formation involves multiple metabolic and regulatory pathways, including carbohydrate metabolism, protein metabolism, and redox homeostasis (Liu et al., 2010; Lin et al., 2014, 2017a,2017b; Biselli et al., 2015; Sera et al., 2019). Many genes in these pathways have been cloned, and their functions have been comprehensively expounded. *WB1* (*white belly 1*) and *GIFI* (*grain incomplete filling 1*) both encode cell-wall invertases, while *GIFI* in wild rice and *wb1* showed enhanced chalkiness (Wang et al., 2008, 2018). *OsSSIIIa/OsFLO5* (*floury endosperm 5*) encode soluble starch synthases *IIIa*, and the *flo5* showed white-core floury in the endosperm (Ryoo et al., 2007; Zhang et al., 2011). In addition, *OsFLO2*, *FLO4*, *OsFLO6*, and *OsFLO13* are all probably involved in the synthesis of starch, and the mutation of each one in these genes could produce floury endosperm (Kang et al., 2005; She et al., 2010; Cheng et al., 2014; Hu et al., 2018). *OsPDIL1-1*, *FLO11/OsHsp70CP2*, and *OsBiP1/OsBIP3* encode protein disulfide isomerase-like enzyme 1-1 (PDIL1-1), 70-kDa plastid-localized heat shock protein 2 (*FLO11/OsHsp70CP2*), and endosperm lumenal binding protein (*OsBiP1/OsBIP3*), respectively (Wakasa et al., 2011; Han et al., 2012; Tabassum et al., 2020), which are involved in protein metabolism. The absence of *OsPDIL1-1* led to the formation of floury endosperm (Han et al., 2012), while severe suppression or significant overexpression of *OsBiP1* resulted in the floury endosperm (Wakasa et al., 2011). *flo11-2* significantly increased chalkiness compared with the wild-strain type at 28°C, but not at 24°C (Tabassum et al., 2020). The cloned genes related to chalkiness usually severely affect the development of endosperm to produce the floury phenotype, and the floury phenotype is easily identified and ruled out in the field. As a result, a very small number of genes are available for chalkiness modification in rice breeding. *Chalk5* (a major QTL for grain chalkiness on chromosome 5) is thought to be a genuine gene among them. *Chalk5* encodes a vacuolar H^+^-translocating pyrophosphatase. Overexpression of *Chalk5* would affect the *in vivo* pH balance in the endosperm, which in turn would affect the synthesis of the proteinoplast and the formation of vesicle-like bodies, thereby producing an air gap and leading to chalk production in grain (Li et al., 2014). Moreover, the *GW2* gene for grain width (GW), which encodes an E3 ubiquitin ligase (Song et al., 2007), and the *qGW7* gene, which encodes a TONNEAU1-recruiting motif protein (Wang et al., 2015), are also thought to potentially affect the chalkiness trait. *GW5*/*qSW5*, a cloned gene, is involved in the brassinosteroid signaling pathway to regulate GW (Shomura et al., 2008; Weng et al., 2008; Liu et al., 2017), while some QTL analyses and GWASs found that some major QTLs for rice chalkiness was close to *GW5*/*qSW5* (Wang et al., 2011; Gao et al., 2014; Qiu et al., 2015, 2017). Although *GW5*/*qSW5* has been cloned for a long time, its gene structure was not identified until 2017, and it was then named *GSE5* (Duan et al., 2017). In the context of this complex trait, a very small number of chalkiness-related genes available for chalkiness modification in rice breeding have been fine mapped or cloned despite a large number of identified QTLs.

In our previous studies, a major QTL for rice chalkiness was identified in the RM598-RM5140 interval on chromosome 5 (Wang et al., 2011; Jiang et al., 2017). In this study, near-isogenic lines (NILs) were developed to fine map this major QTL, transgenic lines were used to confirm the function of the candidate gene, and transcriptome, proteome, and time-course analyses were performed to explore the molecular mechanisms of chalkiness formation.

## Materials and Methods

### Materials and Mapping Populations

**FIGURE 1:**
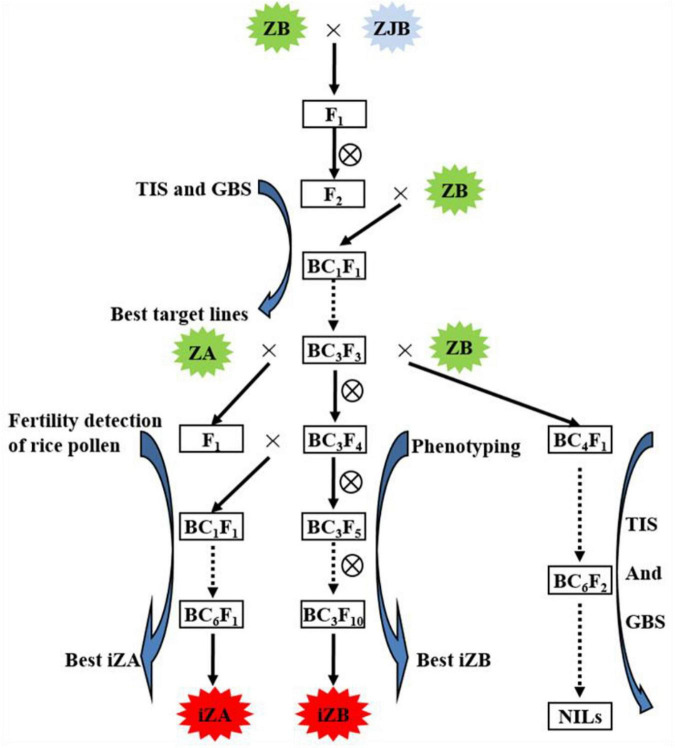
Working routine of the construction of near-isogenic lines (NILs) and iZA breeding by molecular marker-assisted selection. ZB, Zhengshan 97B; ZJB, Zhengjia B; ZA, Zhengshan 97A; TIS, the target interval selection; GBS, the genetic background selection; iZA, improved ZA; iZB, improved ZB. ZB was the recipient parent, and ZJB was the donor parent of the target gene.

Zhenshan 97B (ZB), an *indica* variety, and a maintenance line. Zhenshan 97A (ZA) is the sterile line corresponding to ZB (Table 1). Zhenjia B (ZJB), a maintenance line germplasm with a long grain shape and good quality was developed by molecular marker-assisted selection (MAS) by the authors (Table 1 and Figure 1).

**TABLE 1:** The phenotypes of the parents.

Varieties	GL of milled rice (mm)	GW of milled rice (mm)	PGWC (%)	DEC (%)
Zhenjia B	6.63 ± 0.03	2.10 ± 0.01	1.00 ± 1.00	0.17 ± 0.07
Zhenshan 97B	5.55 ± 0.05	2.60 ± 0.01	77.00 ± 2.00	14.10 ± 1.30

The materials, Zhonghua 11 (ZH11), *proActin:GSE5*, and *gse5-cr* were provided by Professor Yunhai Li (the Institute of Genetics and Developmental Biology, Chinese Academy of Sciences) and Plant Bioscience Limited (Norwich N, England). ZH11 is a *japonica* variety. *proActin:GSE5* is a line with overexpressed *GSE5* in ZH11 background, and *gse5-cr* is a line with a 1-bp deletion mutant of *GSE5* in ZH11 background (Duan et al., 2017).

In this study, NILs (BC_6_S_2_, BC_7_S_2_, and BC_7_S_3_) were constructed based on MAS, in which ZB was the recipient parent, ZJB was the donor parent of the target gene (Figure 1). MAS includes two processes. One is foreground selection, which is target gene or interval selection (TIS), and the other is genetic background selection (GBS), which selects the genetic background from the recurrent parent using the molecular markers showing polymorphism between the parents. In each backcross separation population, such as BC_1_F_1_, BC_2_F_2_, BC_3_F_2_, and so on, the lines with the target interval and the most genetic background of recurrent parents (ZB) were selected for the next backcross. Three pairs of simple sequence repeat (SSR) markers (RM169, RM289, and RM598) were used for TIS and 95 pairs of SSR markers (Supplementary Table 1) showing polymorphism between ZJB and ZB were used for GBS in MAS breeding (Wang et al., 2011).

Sixty-nine rice varieties from around the world (Supplementary Table 2) were provided by the National Seed Bank of China for haplotype analysis of *GSE5*.

### Phenotypic Analysis of Grain Shape and Chalkiness-Related Traits

Rice grains were collected from the plants during the ripening stage. The grains were dried in an oven at 60°C until a constant weight was achieved.

Brown rice was made from rice grains using the hulling machine JLGJ4.5 (Taizhou Grain Instrument Factory, Zhejiang, China). The brown rice was processed to milled rice with a rice milling machine PEARLEST (KETT, Japan). More than 100 whole-grain milled rice grains with normal development were selected and spread on a scanner such that no contact and overlap occurred between the grains. The scanner was connected to a computer, and the JMWT 12 analysis software (Beijing Oriental Fude Technology Development Co. LTD, China and SATAKE MultiMix Corporation, Japan.)^1^ was opened. We selected the standard, edible rice GB1354, and *indica* rice analysis menu and then clicked “Begin” to start the scan. Phenotypic values, including PGWC, DEC, grain length (GL), and GW, were generated.

### Scanning Electron Microscopy Observation of the Endosperm Starch in the Rice Grains

The rice grain was broken at the midpoint of the endosperm longitudinal axis. The cross-section was subjected to spraying with gold for 60 s under 7 Pa of pressure and a 15-mA current. The Hitachi desktop scanning electron microscope TM-1000 (HITACHI Corporation, Japan) was used for scanning and imaging the grains.

### Extraction of Plant Genomic DNA

Genomic DNA was extracted and purified using the cetyltrimethylammonium bromide (CTAB) method (Wang and Fang, 2003).

### Primer Design and Synthesis

The sequences of all the RM series SSR markers can be found on the GRAMENE website^2^. Other primers were self-designed using the Primer Premier 6 software (PREMIER Biosoft, USA)^3^ by referring to the sequences of the rice varieties Nipponbare (*Oryza sativa* subsp. *japonica*) and 9311 (*Oryza sativa* subsp. *indica*). All the primers were synthesized by Xiamen Baijin Biological Technology Co. Ltd. (Xiamen, China).

### Linkage Map Construction for the Quantitative Trait Locus Analysis

In this study, 59 pairs of primers from the RM series SSR on chromosome 5 (Supplementary Table 3) and 17 pairs of self-designed XM series primers (Supplementary Table 4) were used for polymorphism analysis between the parental lines. Polymorphic primers were used for the genotypic analysis of the genetic population and the construction of a genetic linkage map.

Phenotypic and genotypic analyses of 316 NILs (BC_6_S_2_) were performed, and then a linkage map was constructed with the QGAStation 2 software (Zhejiang University, China)^4^ based on the genotypes of these lines. According to the software user’s manual, we first imported molecular marker data, selected the “linkage map” menu, and then set the linkage map construction criteria by selecting “*P* = 0.05” to run the software. The linkage map data was generated upon completion of the computation. Then, QTLNetwork 2.1 was used for QTL mapping (see text footnote 4). First, we created the map file and data file following the user manual, opened QTLNetwork 2.1, clicked “create a new file,” imported the map file and data file, and then clicked “OK.” Next, we set relevant parameters [e.g., set the significance level as 0.05 and the analysis method as Markov chain Monte Carlo (MCMC)]. Finally, we clicked the “Confirm” button to start the analysis, and the results were shown when the analysis was completed.

### Fine Mapping and Gene Sequence Analysis of the Candidate Gene

In fine mapping, 66 pairs of primers (Supplementary Table 5) were synthesized and 37 pairs of SSR primers were self-designed and synthesized (Supplementary Table 6).

Among NILs (BC_6_S_2_), 1,083 lines with high chalkiness (homozygous recessive) were chosen for fine mapping of the candidate gene. Molecular markers in the target interval were used for the analysis of the exchange rate. New PCR primers were designed in a region closer to the target candidate gene according to the previous mapping result and the whole-genome sequences of Nipponbare and 9311. Further analysis of the exchange rate of these molecular markers was used to derive the physical distance between the target gene and each of the molecular markers. Following this procedure, the target gene was mapped to a rather small physical interval. According to the genome annotation in this interval and the target trait, the candidate gene was inferred through the analysis.

For the sequence analysis, 4, 4, 2, 2, 2, 3, 4, 2, and 2 pairs of primers (Table 2) were used in this study to amplify the full-length sequences of the *Chalk5, GS5*, LOC_Os05g09460, LOC_Os05g09470, LOC_Os05g09480, LOC_Os05g09490, LOC_Os05g09500, LOC_Os05g09510, and LOC_Os05g09520 genes, respectively.

**TABLE 2:** Primers for amplifying the full-length sequences of the genes in the target interval, *Chalk5*, and *GS5*.

Gene MSU ID/name	Length of gene (bp)	Primer name	sequence	Position (bp)
LOC_Os05g09460	2265	09460-1F	GCATCTGGACCAACCGATCATG	−1068
		09460-1R	CTTGTGGAGATAGTGCGAGAGGAG	1303
		09460-2F	GGACGGCGGGTGTGAACTTA	672
		09460-2R	TCCCAACCCAATGACTAGGATGG	2256

LOC_Os05g09470	2664	09470-1F	CCACTCGCAGAGCCACTTGTAG	−1174
		09470-1R	GAGGACCAGGTAGTAGCCGTAACT	996
		09470-2F	GACATGATTGCGAGCGGACATC	578
		09470-2R	GCGTGCAGACCTTGGAGTAACT	2873

LOC_Os05g09480	1910	09480-1F	CCAAGAACGGTAATAACGCCTCAG	−1106
		09480-1R	CCTGCCATTCCAAGCCATCTCT	474
		09480-2F	CGCCAGTAATGTTGTTGCTCCTC	−111
		09480-2R	GACCGCTCGGACGAATAACTACA	2080

LOC_Os05g09490	3180	09490-1F	TCTGTGGTATTGTTCGGTTGTTCG	−1678
		09490-1R	TAGGGAGGAGAAGCTAGGGTTGAA	347
		09490-2F	CCCGATGTGATACGCCGAAACT	−167
		09490-2R	GCAGCACTGACATGACCTTGGTA	1815
		09490-3F	GGAACTGCCGCTGACACTGA	1411
		09490-3R	GAAGGAGCCTGATGGACTCTACAA	3680

LOC_Os05g09500	4016	09500-1F	ACTGCCAACCGCACAAGTATTG	−1331
		09500-1R	GTCTCCGACCAGCAACCAATC	+188
		09500-2F	TTCCTCATCTCCTCCTCCTCCTAA	+35
		09500-2R	AACAAGCCCTCTTCGGTCCTAC	+1594
		09500-3F	ACAGAATAGTGGAACAGCAATGTG	+1248
		09500-3R	TGGAAGACAGGATGAGGAGAAGTT	+2499
		09500-4F	AACGCCGCATATCTTGAGAAGG	+2479
		09500-4R	ATAAGGGCAACGATGGGTGAAA	+4090

LOC_Os05g09510	1083	09510-1F	CAGATGACAGGCGAGCGATGAG	−1119
		09510-1R	GCGACGAAGTCTTGAACCTTGGT	146
		09510-2F	CGAGAACCAGCGAGCGAGTA	−283
		09510-2R	AGATGACAGGCGAGCGATGAG	2284

LOC_Os05g09520/*GSE5*	6270	09520-1F	TGTCACCGCCGCAATCCTT	−2583
		09520-1R	AGCCTCTGAATGCCGTCTGGA	+439
		09520-2F	CTGCGGTTTCCCTCTCTTA	−120
		09520-2R	ACTTGTCTTGGTCTCCTTCA	2692

LOC_Os05g06480/*Chalk5*	4570	XMCH5-2F	ACGCCAGCAGGAAACCCAT	+702
		XMCH5-2R	TCCAAATAGAGGAGCCACGACAAG	−1020
		XMCH5-4F	ATCACCAATCCCGCCCACAG	+3040
		XMCH5-4R	TCCAGCACGCCATCTCCGTT	+337
		XMCH5-3F	ATTTGCTCTGTTCTGTTGGTCC	+4626
		XMCH5-3R	TCTGCCCATCGTTCACTTCC	+2131
		XMCH5-1F	TGGTCGGAGGTGAGATAGT	+4916
		XMCH5-1R	GAAGACGATGAGCATTAGGG	+4281

LOC_Os05g06660/*GS5*	4560	XMGS5-1F	TTTCCATCGTTGTCACGCT	−1713
		XMGS5-1R	AGACCTCCATTGAGCCAGA	+462
		XMGS5-2F	CACCAAGAATCGCACCACT	+39
		XMGS5-2R	GTGAAAGAAGCCCAATGTAGC	+2279
		XMGS5-3F	TGTCCAAGATGCCTTCCTTTC	+1622
		XMGS5-3R	TACTCCACAAACCTCCCAGC	+4135
		XMGS5-4F	CCTACCGATCAAGACCGATTGG	+3973
		XMGS5-4R	CCTACTTGCTCGACTTACTGTTGT	+4839

### Real-Time PCR Analysis

In this experiment, 56 pairs of primers of the genes showing different expression levels in our “omics” analysis or are known genes associated with chalkiness were designed and used for qRT-PCR analysis (Supplementary Tables 7, 8). Total RNA was isolated from dehulled kernels with TaKaRa MiniBEST Universal RNA Extraction kits (Takara Bio Inc., China). cDNA synthesis was carried out with the Thermo Scientific RevertAid First Strand cDNA Synthesis Kit (Thermo Fisher Scientific, United States). The actin gene (LOC_Os03g50885) was used as the reference expression gene. SYBR Green II real-time PCR was carried out using the TransStart^®^ Top Green qPCR Super Mix Kit (TransGen Biotech, China) on an ABI Prism 7500 Sequence Detector (Applied Biosystems, Inc., United States). The real-time PCR amplification mixture (20 μl) contained 1 μg of cDNA, 10 μl of 2 × TransStart^®^ Top Green qPCR Super Mix Kit, 0.4 μl of 50 × Dye II and 4 μl of 5 μM forward and reverse primers.

### RNA Sequencing and Analysis

The dehulled developing caryopsis was collected at 10 days after pollination (DAP) and 15 DAP on ZB and NIL^*qDEC*5^ lines (BC_7_S_3_) in the field, and each line had three biological replicates. A total amount of 3 μg RNA per sample was used as input material for RNA sample preparation. RNA-Seq libraries were prepared by the NEBNext^®^ Ultra™ RNA Library Prep Kit for Illumina^®^ (NEB, United States), according to manufacturers’ instructions. The libraries were sequenced on an Illumina HiSeq 2500/X platform (Illumina, United States), and 125/150-bp paired-end reads were generated. Raw data (raw reads) in fastq format were first processed through in-house Perl scripts. At the same time, Q20, Q30, and GC contents of the clean data were calculated. All the downstream analyses were based on clean data with high quality. Reference genome and gene model annotation files were downloaded from the genome website directly.^5^ ^5^ The index of the reference genome was built using Bowtie version 2.2.3 (Johns Hopkins University, USA), and paired-end clean reads were aligned to the reference genome using TopHat version 2.0.12 (Johns Hopkins University, USA) (Trapnell et al., 2009). Cuffquant and cuffnorm (version 2.2.1) were used to calculate fragments per kilobase of transcript per million mapped reads (FPKMs) of genes in each sample (Trapnell et al., 2010). Here, differential expression analysis between two samples, ZB and NIL^*qDEC*5^ at the same developmental stage, was performed using the DESeq2 R package (European Bioconductor Conference, Germany) (Wang et al., 2010). The resulting *P*-values were adjusted using Benjamini and Hochberg’s approach for controlling the false discovery rate. Genes with an adjusted *P*-value < 0.01 found by DESeq2 were assigned as differentially expressed. The sequencing data were submitted to GenBank (PRJNA751381).

### Gene Ontology Term Enrichment Analyses

The GOseq R package was employed to analyze Gene ontology (GO) enrichment since it was developed to account for RNA length bias typical of RNA-Seq approaches (Young et al., 2010). GO terms with corrected *P*-value < 0.05 were considered significantly enriched by differentially expressed genes (DEGs).

### Protein Extraction, Two-Dimensional Electrophoresis, and Image Acquisition

The dehulled developing caryopsis was collected at 6 DAP, 12 DAP, 18 DAP, and 24 DAP from ZB, ZJB, NIL^*qDEC*5^, and NIL^*qDEC*5^ lines (BC_7_S_2_) in the field, and each line had three biological replicates. The methods of protein extraction, two-dimensional electrophoresis, and image acquisition refer to the paper (Chen et al., 2014).

### Mass Spectrometry Analysis and Database Search

The selected protein spots were excised from the 2-DE gels and digested with sequencing-grade trypsin (Promega Corporation, United States). The method of mass spectrometry analysis and database searches for the peptides refer to the paper (Chen et al., 2014).

### Protein-Protein Interaction Analysis

Protein-protein interaction (PPI) analysis of DEGs was based on the STRING database^6^.

### Heat Shock Treatment

In the research field, three lines (3 plants/lines), *proActin:GSE5*, ZH11, and *gse5-cr*, planted in square plastic pots (45 cm × 60 cm) and grown under natural conditions (from August to November in Xiamen, Fujian, China). At 3 DAP, the pots were moved to an illumination incubator with a double-temperature zone (MPI-1008HF-2R, Ningbo Jiangnan Instrument Factory, Ningbo, China) and grown until the rice grains were ripe. One zone is the control condition (28°C, 100,000 Lux for 12 h/26°C, dark for 12 h), while the other zone is a high-temperature condition (34°C, 100,000 Lux for 12 h; 30°C, dark for 12 h). At 6 and 12 DAP, the developing caryopsis was detached from the panicles, immediately frozen in liquid nitrogen, and stored at −80°C until the determination of transcript levels. At 30 DAP, the mature seeds were harvested and used for phenotyping after drying in an oven at 60°C.

### Molecular Marker-Assisted Selection and Breeding

In the previous study, our research group found that the RM598-RM169-RM289 interval of chromosome 5 had a strong influence on chalkiness and grain shape (Wang et al., 2011). In this study, MAS was used to rapidly improve the maintainer line, ZB, and its corresponding sterile line, ZA. In this study, ZJB was the donor of high-quality genes corresponding to rice appearance quality traits and ZB was the receptor. In F_2_ from the hybridization between ZJB and ZB, TIS and GBS were carried out using molecular markers, and then the selected lines were backcrossed with ZB. After self-crossing, BC_1_F_2_ was obtained, and then TIS and GBS were carried out again. The selected lines continued to backcross, the BC_3_F_2_ population was finally produced, and MAS was carried out again to obtain iZB (Figure 1). Three SSR markers, RM598, RM169, and RM289, were used for TIS. Ninety-five pairs of SSR markers showing polymorphism between ZB and ZJB were used for GBS (Supplementary Table 1).

### Method for Calculating the Homozygosity Degree

In this study, the homologous degree (HD) indicates that the recurrent parent genotype (genetic background) accounts for a proportion of the entire rice genome in the progeny individuals. HD was calculated based on the formula 1-*ax* (*a*, the polymorphism between ZB and ZJB, *a* = 16.37%) (Wang et al., 2011); *x* refers to the genetic polymorphism rate in the intervals of genetic background selection, and *x* is the number of polymorphic markers between the selected lines and ZB/the number of the total markers used for GBS.

### Fertility Identification of Rice Pollen Granules by Microscope

The fertility of rice pollen granules was identified by microscopy. After rice pollen granules were impregnated with 1% iodine/potassium iodide aqueous solution (I_2_/KI), the fertile pollens showed complete black solid round grains, which were called “stained black,” and the sterile pollens were light-colored and round or irregularly shaped, which were called “light stained.”

At approximately 8:00 a.m., spikes that were about to blossom were picked from rice plants and placed into a plastic bag for moisturization. A drop of 1% I_2_/KI was dropped on a glass slide. The glume was peeled off with tweezers, and all the six anthers on every spikelet were taken out and placed in the 1% I_2_/KI on the glass slide for staining. Then, the cover glass was placed over the solution and gently pressed to release the pollen granules from the broken pollen sac. They were then observed under a light microscope with 10 × 10 magnification. Two spikelets were taken from each panicle every time, three fields were observed, and each field was counted as 300 pollen granules. Two replicates were applied for each panicle.

## Results

### Mapping of *qPGWC5* and *qDEC5*

**FIGURE 2:**
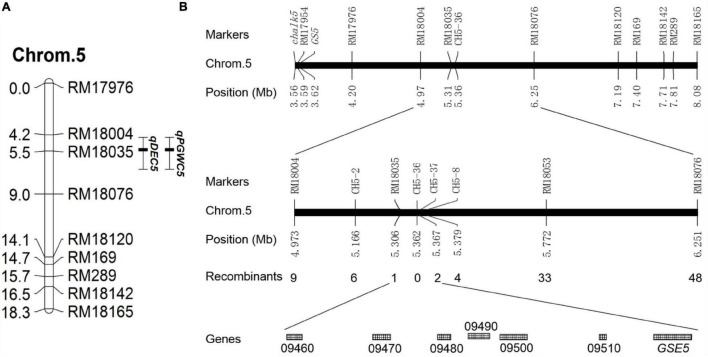
Genetic linkage map and physical map. **(A)** Genetic linkage map. Chrom.5 indicates chromosome 5, the numbers on the left side are the genetic distances (cM), the numbers on the right side are the molecular marker names, *qPGWC5* is a QTL for PGWC, and *qDEC5* is a QTL for DEC. **(B)** Fine mapping of *qDEC5*. “LOC_Os05g + the number” shown on the last line indicates the gene number in the rice gene annotation library of the Michigan State University (MSU) website (http://rice.plantbiology.msu.edu/).

To locate the causal interval for chalkiness traits on chromosomes, a linkage group was constructed on chromosome 5 based on the BC_6_S_2_ population. The QTL analysis showed that a QTL for PGWC (*qPGWC5)* and a QTL for DEC (*qDEC5*) were colocalized and mapped to the RM18004—RM18035 interval on chromosome 5. The additive effect and the genetic contribution rate [H^2^_(A)_] of these two QTLs were 20.49 and 47.2% and 3.31 and 40.7%, respectively (Table 3 and Figure 2A). These results indicated that one major QTL for the chalkiness trait was located in the RM18004—RM18035 interval, which was named *qDEC5*.

**TABLE 3:** Major QTLs for the chalkiness trait in the RM18004—RM18035 interval.

Name of the QTL	A*^a^*	H^2^_(A)_*^b^* (%)	*P*-Value
*qPGWC5*	20.49	47.2	0.000
*qDEC5*	3.31	40.7	0.000

*^a^Additive effect.*

*^b^Heredity variance (H^2^) explained by the QTL.*

### Fine Mapping of *qDEC5* Using Near-Isogenic Lines

A total of 1,083 NILs with high chalkiness (homozygous recessive) and 37 pairs of self-designed primers (CH5 series) were used to obtain recombinants in the target interval. *qDEC5* was mapped in the interval of 61 kb between RM18035 and CH5-37 (Figure 2B and Supplementary Table 9), harboring seven known genes (LOC_Os05g09460, LOC_Os05g09470, LOC_Os05g09480, LOC_Os05g09490, LOC_Os05g09500, LOC_Os05g09510, and *GSE5*) (Figure 2B and Supplementary Table 10).

### *GSE5* Was the Candidate Gene of *qDEC5*

To screen for the candidate gene, sequence analysis of the seven genes and their leading sequences in the interval was carried out. The result indicated no differences in six of the genes between the two parental lines (LOC_Os05g09460, LOC_Os05g09470, LOC_Os05g09480, LOC_Os05g09490, LOC_Os05g09500, and LOC_Os05g09510) (Table 2). *Chalk5* for rice chalkiness (Li et al., 2014) and *GS5* for rice grain shape (Li et al., 2011), are also very close to *GSE5* on chromosome 5. The sequence analysis of these two genes showed no difference between the two parental lines (Table 2). However, differences were detected in *GSE5*.

**FIGURE 3:**
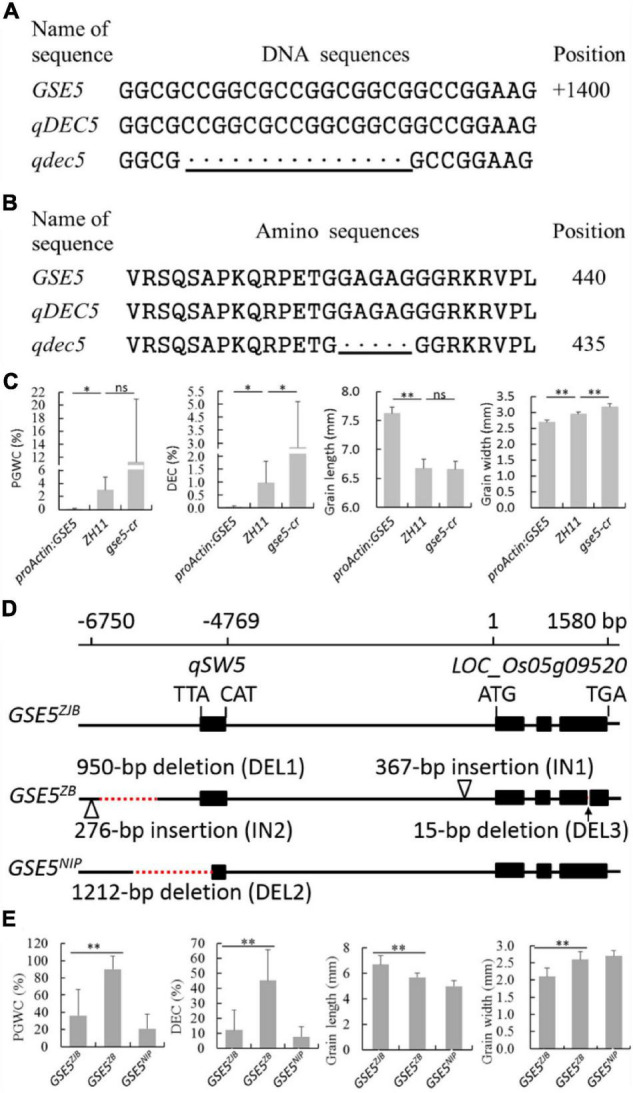
Genotyping and phenotyping analysis of the *GSE5*. **(A)** DNA sequence alignment of *qDEC5* between ZB and ZJB. **(B)** Amino sequence alignment of *qDEC5* between ZB and ZJB. **(C)** Phenotyping analysis of ZH11, *proActin:GSE5* and *gse5-cr*. **(D)** The three major haplotypes of *GSE5* (*GSE5^ZJB^*, *GSE5^ZB^*, and *GSE5^NIP^*). ZJB, ZB, and NIP are Zhenjia B, Zhenshan 97B, and Nipponbare, respectively. **(E)** The phenotyping analysis of the rice varieties with different haplotypes. The bottom line shows the mutations. Every black “.” indicates a one-base or one-amino acid deletion. Significance is determined using analysis of variance (ANOVA) (*P < 0.05, ^**^P < 0.01, and ns represents no signification). The red dashed lines represent deletions, and the triangles represent insertions in the genomic regions.

Sequence analysis of *GSE5* indicated a 15-bp deletion from 1,372 to 1,386 bp in ZB, the parent with high chalkiness (Figure 3A and Table 2). The deletion was at the third exon and resulted in a deletion of five amino acids covering positions No. 428 to No. 432 in the encoded protein (Figure 3B).

### *GSE5* Regulates Chalkiness and Grain Shape

The *proActin:GSE5* transgenic line and *gse5-cr* mutant generated by CRISPR/Cas9 with the genetic background of ZH11 were planted to confirm the function of *qDEC5*. Phenotyping and Student’s *t*-test showed that PGWC and DEC were markedly lower in *proActin:GSE5* lines than in ZH11, while DEC was markedly higher in the *gse5-cr* mutant than in ZH11 (Figure 3C). GL in *proActin:GSE5* was extremely significantly longer than in ZH11, while GW was extremely significantly smaller (Figure 3C). GW was extremely significantly greater in *gse5-cr* than in ZH11, whereas GL was similar to that in ZH11 (Figure 3C). These results indicate that *qDEC5* controls rice chalkiness and grain size.

We identified three major haplotypes of *GSE5* (*GSE5^ZJB^*, *GSE5^ZB^*, and *GSE5^NIP^*) in 69 rice varieties from around the world (Figures 3D,E). Among them, thirty-nine *indica* varieties with the *GSE5^ZJB^* haplotype showed a lower PGWC and DEC and a slim grain shape (Figure 3E and Supplementary Tables 2, 11). Another 17 *indica* varieties with the *GSE5^ZB^* haplotype showed a higher PGWC and DEC and a shorter and wider grain shape (Figure 3E and Supplementary Tables 2, 11). The *GSE5^ZB^* haplotype contains a 950-bp deletion (DEL1), a 276-bp insertion (IN2) in the 3′ flanking region of *qSW5*, a 367-bp insertion (IN1) in the flanking region of LOC_Os05g09520, and a 15-bp deletion (DEL3) in the third exon of LOC_Os05g09520 (Figure 3D). Thirteen *japonica* varieties had the *GSE5^NIP^* haplotype, which contains a 1212-bp deletion (DEL2) in *qSW5* and its 3′ flanking region (Figure 3D).

### *GSE5* Is a Semi-Dominant Gene Regulating Chalky Trait

**FIGURE 4:**
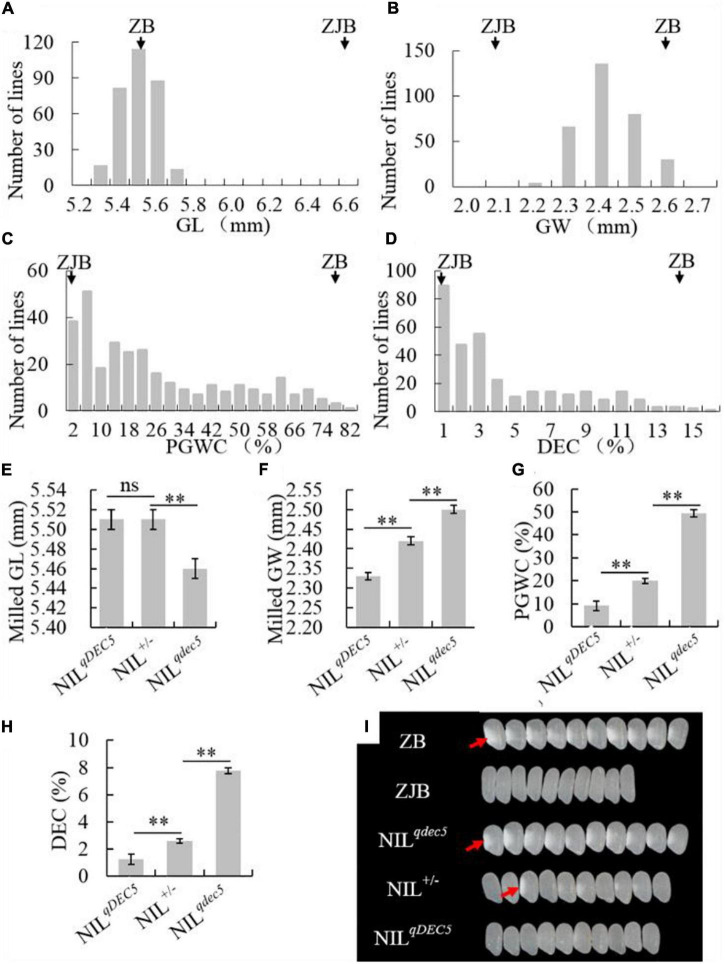
The phenotype and genetic characteristics of grain shape and chalkiness traits. The distribution of the GL **(A)**, GW **(B)**, PGWC **(C)**, and DEC **(D)** traits in the NIL population. The average GL **(E)**, GW **(F)**, PGWC **(G)**, and DEC **(H)** values for three different groups of NILs (NIL^*qDEC*5^, NIL^±^, and NIL^*qdec*5^), where ZJB and ZB represent Zhenjia B and Zhenshan 97B, respectively, ** denotes a difference at a very significant level, and ns represents no signification. **(I)** Images of milled rice grains of ZB, ZJB, and the three different types of NILs, where the white parts (red arrows) represent rice chalkiness.

To study the genetic characteristics of *GSE5*/*qDEC5*, milled rice grain shape and chalkiness phenotypic analysis of ZB, ZJB, and the NIL population (BC_6_S_2_, 316 lines) was carried out. The milled rice GL, GW, PGWC, and DEC values for ZB were 5.55 ± 0.05 mm, 2.6 ± 0.01 mm, 77 ± 2%, and 14.1 ± 1.3%, respectively, whereas the values for ZJB were 6.63 ± 0.03 mm, 2.1 ± 0.01 mm, 1 ± 1%, and 0.17 ± 0.07%, respectively (Table 1). For the NILs, the milled rice GL and GW were in a nearly normal distribution (Figures 4A,B), and the distributions of the PGWC and DEC showed a nearly three-peak distribution (Figures 4C,D).

The NILs were divided into three groups based on the *GSE5*/*qDEC5* genotype. The first group (NIL^*qDEC*5^) included 89 lines whose *qDEC5* genotype was from the ZB background. This group had milled rice GL, milled rice GW, PGWC, and DEC values of 5.46 ± 0.01 mm, 2.5 ± 0.01 mm, 49.48 ± 2.13%, and 7.76 ± 0.39%, respectively. The second group (NIL^*qDEC*5^) included 76 lines whose *qDEC5* genotype was from the ZJB background. This group had milled rice GL, milled rice GW, PGWC, and DEC values of 5.51 ± 0.01 mm, 2.33 ± 0.01 mm, 9.07 ± 1.58%, and 1.23 ± 0.24%, respectively. The third group (NIL^±^) included 151 lines whose *qDEC5* genotype was heterozygous. This group had milled rice GL, milled rice GW, PGWC, and DEC values of 5.51 ± 0.01 mm, 2.42 ± 0 mm, 19.97 ± 1.04%, and 2.61 ± 0.16%, respectively (Figures 4E–H and Supplementary Table 12).

The milled rice GL was the same for the NIL^±^ and NIL^*qDEC*5^ lines but significantly different from the NIL^*qDEC*5^ lines. For the milled rice GW, PGWC, and DEC traits, the average values of each trait for the NIL^±^ group were significantly different from those of the other two groups (the NIL^*qDEC*5^ and NIL^*qDEC*5^ lines), as were the median values of the NIL^*qDEC*5^ and NIL^*qDEC*5^ lines (Figures 4E–I and Supplementary Table 13). The above results showed that *qDEC5* controls both the grain shape and chalkiness traits, and the milled rice GL trait exhibits dominant inheritance, while the milled rice GW, PGWC, and DEC traits exhibit semi-dominant inheritance.

### Scanning Electron Microscopy Observations of the Endosperm Starch in Rice Grains

**FIGURE 5:**
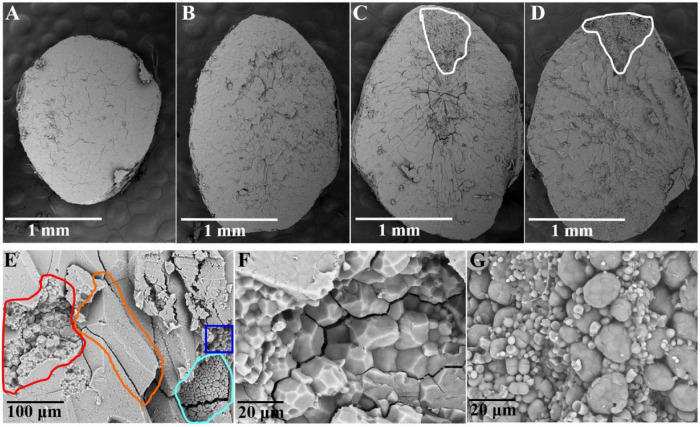
Transverse sections of the rice endosperm observed by SEM. Shown in panels **(A–D)** are whole transverse sections of a ZJB, NIL^*qDEC*5^, NIL^*qdec*5^, and ZB rice grains, respectively, as observed by SEM. The chalky part is located within the white circle **(C,D)**. Panel **(E)** is a local cross-section of a ZB rice grain. The orange circle indicates one side of a whole starch cell in the non-chalky part, the red circle indicates the torn cell cross-section in the non-chalky part, the blue circle indicates one side of the whole cell in the chalky part, and the blue-purple circle indicates the torn cell cross-section in the chalky part. **(F)** Magnified image of some parts of the red circle in panel **(E)**. **(G)** Magnified image of some parts of the blue-purple circle in panel **(E)**.

Scanning electron microscopy (SEM) observations of the transverse sections of whole-grain rice were carried out to understand the cell contour in the matured endosperm. In the non-chalky ZJB variety and the NIL^*qDEC*5^ lines, the transverse section appeared quite neat and smooth (Figures 5A,B). In the high-chalkiness ZB variety and the NIL^*qDEC*5^ lines, the non-chalky region had a pattern similar to the non-chalkiness lines, but the chalky part was granular throughout the whole region with an unclear cell contour (Figures 5C,D).

For ZB, upon zooming in the local cross-section, the cell surface in the non-chalky part was smooth, and the cell had a polyhedral and angular shape, whereas the cell surface in the chalky part had a smoothly arched shape (Figure 5E). The transverse sections of torn cells were enlarged 2000 times for detailed observation. In the non-chalky part, the amyloplasts were rather uniform in size, polyhedral and angular in shape, and tightly packed, and very few exposed amyloplasts can be seen (Figure 5F). In contrast, in the chalky part, the amyloplasts were round or oval in shape and loosely packed. The large round granule (amyloplast) is composed of many small round granules, which are most likely exposed starch granules (Figure 5G). Thus, the most pronounced structural differences between the chalky and non-chalky parts of the endosperm were the composition and arrangement of the amyloplasts.

### Time-Course Analysis of Some Genes Related to Chalkiness or Starch Biosynthesis During the Process of Seed Development by Quantitative Real-Time-PCR

**FIGURE 6:**
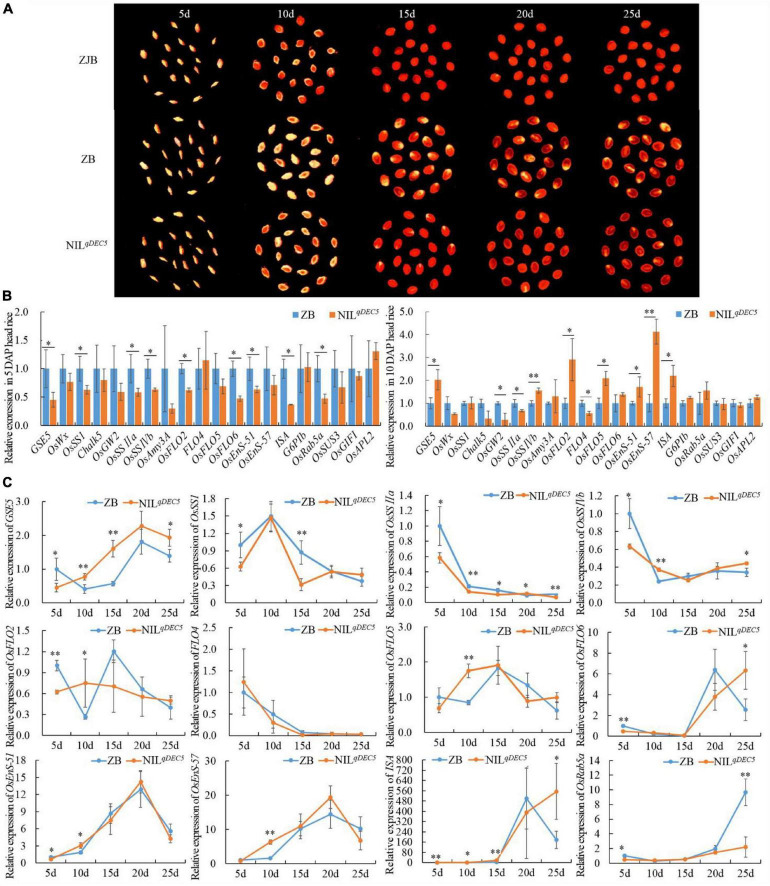
The dehulled developing caryopsis and the relative expression of *GSE5* and genes related to chalkiness or starch metabolism during the process of seed development. **(A)** The dehulled caryopsis cross-section of ZJB, ZB, and NIL^*qDEC*5^ at different developmental stages was obtained by the negative scan function of the HP ScanJet G4050 scanner. The yellowish-white spot is the floury or chalky part in the endosperm. **(B)** The relative expression levels of genes related to chalkiness or starch biosynthesis during the process of seed development. ZB expression represents 1. **(C)** Time course analysis of genes’ expression during the process of seed development. ZB expression at 5 DAP represents 1. Significance was determined by analysis of variance (ANOVA) (*P < 0.05 and ^**^P < 0.01). Error bar shows the SD (*n* = 3); DAP, day after pollination.

To investigate whether some genes related to chalkiness or starch synthesis were regulated by *GSE5*/*qDEC5*, time-course experiments of 20 genes were performed at 5-time points (5, 10, 15, 20, and 25 days after pollination) during the process of seed development by means of quantitative real-time PCR (qRT-PCR) (Figure 6A and Supplementary Table 7). At 5 DAP, the expression levels of *GSE5*, *OsSSI*, *OsSSIIa*, *OsSSIVb*, *OsFLO2*, *OsFLO6*, *OsEnS-51* (*Oryza sativa endosperm-specific gene 51*), *ISA* (*Oryza sativa isoamylase*), and *OsRab5a* (*small GTPase Rab5*) were lower in NIL^*qDEC*5^ lines with the *GSE5^ZJB^* haplotype than in ZB with the *GSE5^ZB^* haplotype, while the expression levels of *OsWx* (*Oryza sativa waxy*), *Chalk5*, *OsGW2*, *OsAmy3A* (*Oryza sativa alpha-amylase isozyme 3A*), *FLO4*, *OsFLO5*, *OsEnS-57*, *G6PIb* (*glucose-6-phosphate isomerase, cytosolic b*), *OsSUS3* (*Oryza sativa rice sucrose synthase 3*), *OsGIFI* (*Oryza sativa grain incomplete filling 1*), and *OsAPL2* (*Oryza sativa ADP-glucose pyrophosphorylase large subunit 2*) showed no difference between the two haplotypes, and no upregulated gene was found (Figure 6B). At 10 DAP, the expression levels of *GSE5*, *OsSSIVb*, *OsFLO2*, *OsEnS-51*, and *ISA* increased in NIL^*qDEC*5^ and exceeded those in ZB, and only *OsSSIIa* still showed lower expression; the expression levels of *OsGW2* and *FLO4* decreased, while that of *OsEnS-57* markedly increased in NIL^*qDEC*5^ (Figure 6B).

Twelve of the 20 genes showed different expression levels between ZB and NIL^*qDEC*5^ at 5 DAP or 10 DAP, and then the expression dynamics of these 12 genes were processed at 5-time points during the process of seed development. At the same time, two main expression patterns were found in the *GSE5^ZJB^* and *GSE5^ZB^* haplotypes. The first pattern was that the expression levels of *GSE5*, *OsFLO6*, *OsEnS-51*, *OsEnS-57*, and *ISA* showed a trend from low to high during the first 20 days of seed development; then, those of *GSE5*, *OsEnS-51*, and *OsEnS-57* decreased quickly (Figure 6C). At 5 DAP, the expression level of *GSE5* in ZB was significantly higher than in NIL^*qDEC*5^; however, those at 10, 15 and 25 DAP were significantly or extremely significantly lower. The expression levels of *OsFLO6* and *ISA* at 25 DAP decreased rapidly in ZB, but in NIL^*qDEC*5^, their expression levels continued to increase. The second pattern was that *OsSSI*, *OsSSIIa*, *OsSSIVb*, and *FLO4* showed a trend with high expression levels in the early stage of seed development and low levels in the following stage. The expression levels of *OsSSIIa* at 5, 10, 15, and 25 DAP in NIL^*qDEC*5^ were all lower than those in ZB (Figure 6C).

### Transcriptomics Analysis of Dehulled Developing Caryopsis

**FIGURE 7:**
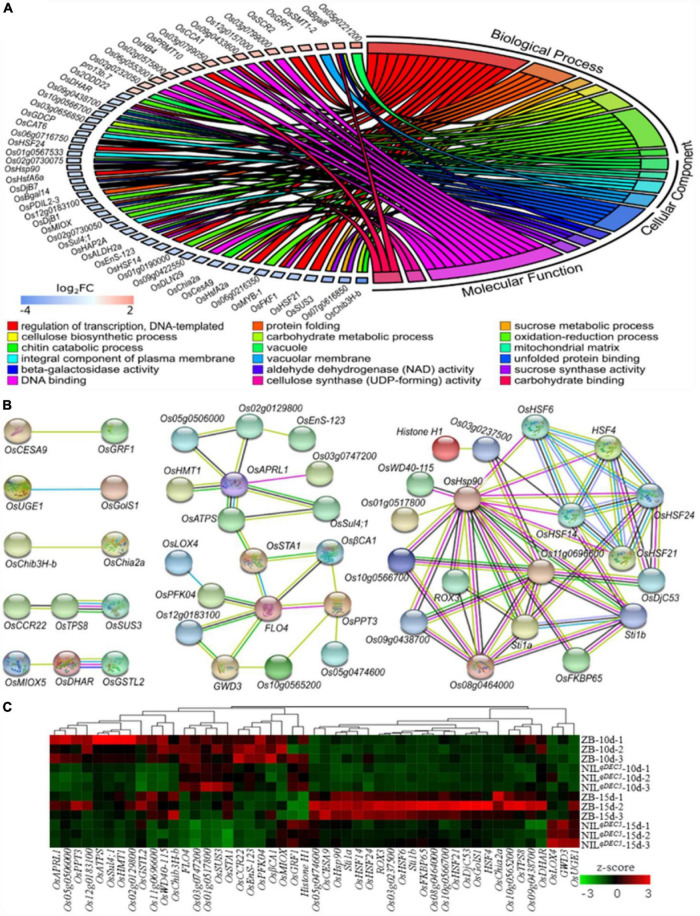
DEGs from the transcriptomic analysis. **(A)** GO enrichment results of DEGs. **(B)** The interaction network of DEGs. DeepSkyBlue1 line, from curated databases; DeepPink3 line, experimentally determined; Green line, gene neighborhood; Blue line, gene co-occurrence; OliveDrab3 line, text mining; black line, co-expression; MediumSlateBlue line, protein homology. **(C)** Hierarchical clustering analysis heat map of the average relative expression levels.

Transcriptional profiles facilitate studies of regulatory networks related to chalkiness in the developing caryopsis. Comparative RNA-Seq-based transcriptomics analysis of dehulled developing caryopsis was conducted at 10 DAP and 15 DAP on ZB and NIL^*qDEC*5^ (BC_7_S_3_). This study revealed 194 and 132 DEGs at the two developmental stages, respectively. The DEGs were involved in various biological processes (regulation of transcription, protein folding, sucrose metabolic process, cellulose biosynthetic processes, carbohydrate metabolic process, oxidation-reduction process, and chitin catabolic process), cellular components (vacuole, mitochondrial matrix, integral component of plasma membrane, and vacuolar membrane) and molecular functions [unfolded protein binding, beta-galactosidase activity, aldehyde dehydrogenase (NAD) activity, sucrose synthase activity, DNA binding, cellulose synthase (UDP-forming) activity, and carbohydrate-binding] based on GO enrichment analysis (Figure 7A). According to the Kyoto Encyclopedia of Genes and Genomes (KEGG) database, the most abundant category was classified as being involved in carbon metabolism (31.7 and 21% of the total annotated DEGs at 10 and 15 days, respectively). The second most abundant class consisted of genes related to amino acid metabolism and protein synthesis, assembly, and degradation (14.6 and 14.5% of the total annotated DEGs at 10 and 15 days, respectively).

Protein-protein interaction analysis was carried out based on the STRING database to develop the interaction networks between the DEGs. The interaction networks can be mainly divided into two groups (Figure 7B). The 29 genes in the first group are probably involved in carbohydrate metabolisms, such as *GWD3* (similar to *Phosphoglucan, water dikinase*), *FLO4*, *OsSTA1* (*putative phosphoenolpyruvate carboxylase*), *OsPFK04* (*pyrophosphate-dependent phosphofructo-1-kinase, phosphofructokinase 4*), *OsCESA9* (*cellulose synthase A catalytic subunit 9*), *OsSUS3*, *OsUGE1* (*UDP-glucose 4-epimerase 1*), and *OsMIOX* (*myo-inositol oxygenase*). *GWD3* mediates the incorporation of phosphate into starch-like phospho-alpha-glucan and may be required for starch degradation. *FLO4* participates in starch metabolism and modulation of carbon flow for starch and lipid biosynthesis during grain filling. *OsSTA1*, *OsPFK04*, *OsCESA9*, *OsSUS3*, and *OsUGE1* are involved in different pathways of carbohydrate metabolism. *OsMIOX* encodes an inositol oxygenase and is involved in the biosynthesis of UDP-glucuronic acid (UDP-GlcA). The 19 genes in the second group are involved in heat stress, such as heat stress transcription factors (*HSF4*, *OsHSF6*, *OsHSF14*, *OsHSF21*, and *OsHSF24*) and heat shock proteins (*OsHSP90*, *OsDjC53*, Os08g0464000, and Os11g0696600).

A hierarchical clustering analysis heat map of the average relative expression levels (log2 ratio) of 49 genes in the gene interaction net was generated. The results showed four main clusters; the genes, including *OsAPRL1* (*adenosine 5′-phosphosulfate reductase-like protein 1*), *OsGSTL2* (*lambda class glutathione S-transferase 2*), and Os11g069660, in the first cluster expressed lower in NIL^*qDEC*5^ at both 10 and 15 DAP, those (including *FLO4*, *OsSUS3*, and *OsPFK04*) in the second cluster were highly expressed at 10 DAP and weakly expressed at 15 DAP; those (such as *OsHSP90*, *OsHSF14*, and *OsDjC53*) in the third cluster showed high expression in ZB at 15 DAP; and the three genes (*OsLOX4*, *GWD3*, and *OsUGE1*) in the fourth cluster were highly expressed only in NIL^*qDEC*5^ at 15 DAP (Figure 7C). Most genes related to heat shock proteins and heat shock transcription factors were clustered in the third cluster (Figure 7C).

### Proteomics Analysis of Dehulled Developing Caryopsis

**FIGURE 8:**
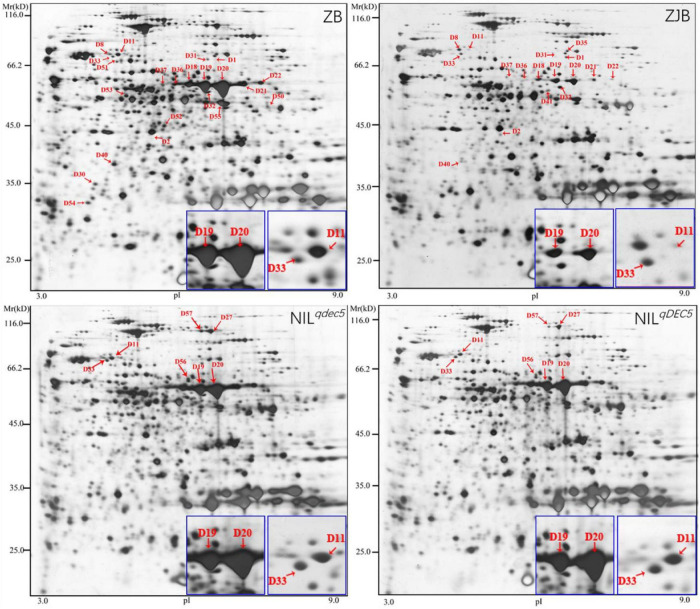
2-DE maps at 12 DAP. Red arrows indicate the positions of protein spots showing differential expression. The blue box is an enlarged view of the corresponding protein spots in the maps.

**FIGURE 9:**
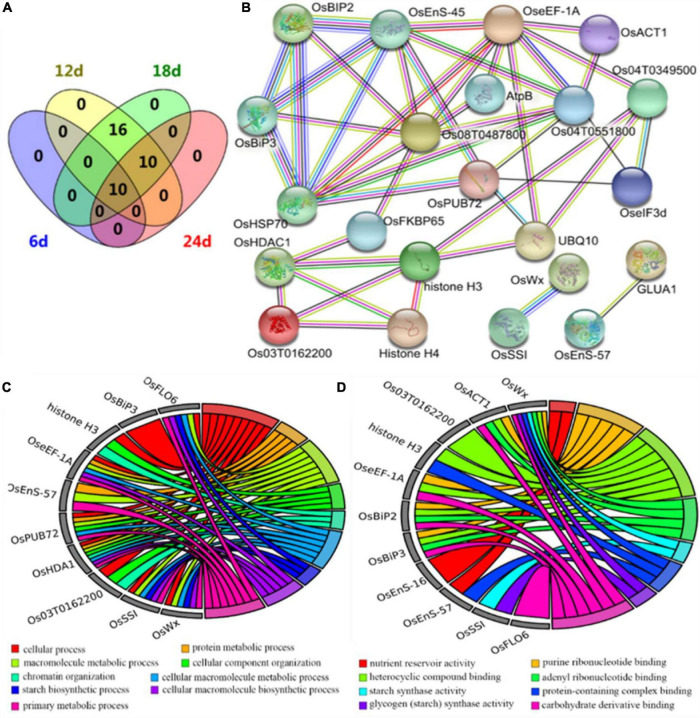
Differentially expressed proteins from proteomics analysis. **(A)** Venn diagram of differentially expressed proteins. **(B)** The interaction network of differentially expressed proteins. DeepSkyBlue1 line, from curated databases; DeepPink3 line, experimentally determined; Green line, gene neighborhood; Red line, gene fusions; Blue line, gene co-occurrence; OliveDrab3 line, text mining; black line, co-expression; MediumSlateBlue line, protein homology. **(C)** Biological process enrichment map from GO analysis. **(D)** Molecular function enrichment map from GO analysis.

To further understand the protein profiles in connection with chalkiness formation in the developing caryopsis, two-dimensional electrophoresis (2-DE) and MALDI-TOF/MS analysis were conducted in rice dehulled developing caryopsis of ZB, ZJB, NIL^*qDEC*5^, and NIL^*qDEC*5^ (BC_7_S_2_) at 6, 12, 18, and 24 days. Approximately 800 protein spots were repeatedly detected in every 2-DE map (Figure 8). Four protein spots (D11, D33, D19, and D20) showed differences both between the parents (ZB and ZJB) and between the two NILs (NIL^*qDEC*5^ and NIL^*qDEC*5^) at the same time. D11 was found in the four stages, D33 was found in the late three stages, and D19 and D20 were found in the middle two stages (Figure 8). These four spots were successfully identified as 36 differentially expressed proteins (DEPs), mainly including the proteins related to starch synthase (OsSSI, OsFLO6, and OsWx) and heat shock (OsHSP70, OsBiP2, OsBiP3, and OS08T0487800) (Figures 9A,B). OsSSI, OsFLO6, and OsWx are a starch synthase I, a CBM48 domain-containing protein, and a granule-bound starch synthase 1, respectively, all of which are involved in starch biosynthesis. These 36 proteins are involved in various biological processes (such as starch biosynthetic process, cellular process, protein metabolic process, and chromatin organization) (Figure 9C), cellular components (such as amyloplast, cell, chloroplast, and intracellular membrane-bounded organelle), and molecular functions [such as starch synthase activity, glycogen (starch) synthase activity, carbohydrate derivative binding, and heterocyclic compound binding] (Figure 9D).

### Differentially Expressed Genes Associated With Carbohydrate Metabolism

**FIGURE 10:**
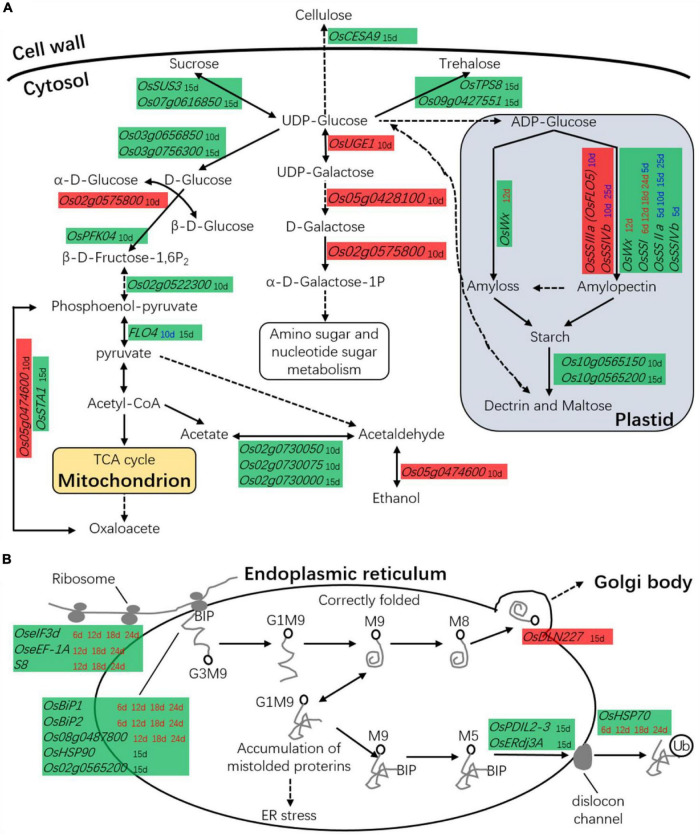
The DEGs in carbon or protein metabolism. **(A)** DEGs are associated with carbohydrate metabolism. **(B)** DEGs are associated with protein metabolism. Red background box, the gene was upregulated in low-chalkiness lines; Green background box, the gene was downregulated in low-chalkiness lines; The time spots in different colored letters indicate DEGs from different sources; Black font, the DEGs from transcriptomic analysis; Red font, the DEGs from proteomics analysis; Blue font, the DEGs from time-course analysis by qRT-PCR. Solid lines represent one-step reactions, dotted lines represent multistep reactions, and arrows denote the reaction direction.

The low-chalkiness lines showed higher amino sugar and nucleotide sugar metabolism at 10 DAP and lower carbohydrate metabolism at 15 DAP. In the above omics analysis, 27 DEGs related to carbohydrate metabolism were identified. These genes were directly involved in starch synthesis and hydrolysis, cell wall biogenesis, glycolysis, pentose phosphate pathway, monosaccharide and oligosaccharide synthesis, gluconeogenesis, and the TCA cycle (Figure 10A). On the other hand, 22 DEGs were identified in the transcriptomic analysis, two in the proteomics analysis, and five in the time course analysis by qRT-PCR.

Transcriptomic analysis showed from UDP-Glucose to α-D-Galactose-1P, *OsUGE1*, Os05g0428100, and Os02g0575800 were upregulated in the low-chalkiness lines at 10 DAP. In other monosaccharide and oligosaccharide synthesis, most DGEs, including *OsSUS3*, *OsPFK04*, *OsTPS8*, *Os07g0616850, Os03g0656850, Os03g0756300*, and *Os09g0427551*, showed low expression at 10 or 15 DAP. *OsCESA9*, which is involved in cellulose synthesis, also showed low expression at 15 DAP. *OsFLO4*, which is involved in conversion between phosphoenol-pyruvate and pyruvate, and *OsSTA1*, which is involved in conversion between phosphoenol-pyruvate and oxaloacetate, were downregulated in the transcriptomic analysis at 15 DAP. Proteomics analysis showed that in starch synthesis, *OsWx* at 12 DAP and *OsSSI* at 6, 12, 18, and 24 DAP was downregulated in the low-chalkiness lines. Time course analysis by qRT-PCR showed that *OsSSI* (at 5 and 15 DAP), *OsSSIIa* (at 5, 10, 15, and 25 DAP), and *OsSSIVb* (at 5 DAP), which are all related to soluble starch synthase, were also downregulated, while *OsSSIIa* (at 10 DAP) and *OsSSIvb* (at 10 and 25 DAP) were upregulated in the low-chalkiness lines.

### Differentially Expressed Genes Associated With Protein Synthesis, Assembly, and Degradation

The low-chalkiness lines showed lower protein metabolism but higher protein transport ability from the endoplasmic reticulum (ER) to the Golgi. In proteomics analysis, seven DEGs associated with protein synthesis, assembly, and degradation were identified and showed downregulation in the low-chalkiness lines (Figure 10B). KEGG analysis showed that *OseIF3d* (*eukaryotic translation initiation factor 3 subunit D*), *OseEF-1A* (*elongation factor 1-alpha*), and *S8* (*40S ribosomal protein S8*) were involved in protein synthesis, while *Os08g0487800* (*similar to heat-shock protein precursor*), *OsBiP1* (*endosperm luminal binding protein 1*) and *OsBiP2* were involved in protein recognition by luminal chaperones. In the transcriptomic analysis at 15 DAP, five DEGs were associated with protein synthesis, assembly, and degradation (Figure 10B). *OsHSP90* (*heat-shock protein 90.1*) and *Os02g0565200* (*signal peptidase complex subunit 2*), which are involved in protein recognition, and *OsPDIL2-3* (*protein disulfide-isomerase 2-3*), *OsERdj3A* (*ER-resident J-protein 3A*), and *OsHSP70* (*heat-shock protein 70*), which are involved in protein degradation, were downregulated in the low-chalkiness lines. *OsDLN227* (*DLN motif protein 227*), which may be involved in ER-Golgi transport, was found to be upregulated.

### Response of Chalkiness and Related Genes to Heat Shock

**FIGURE 11:**
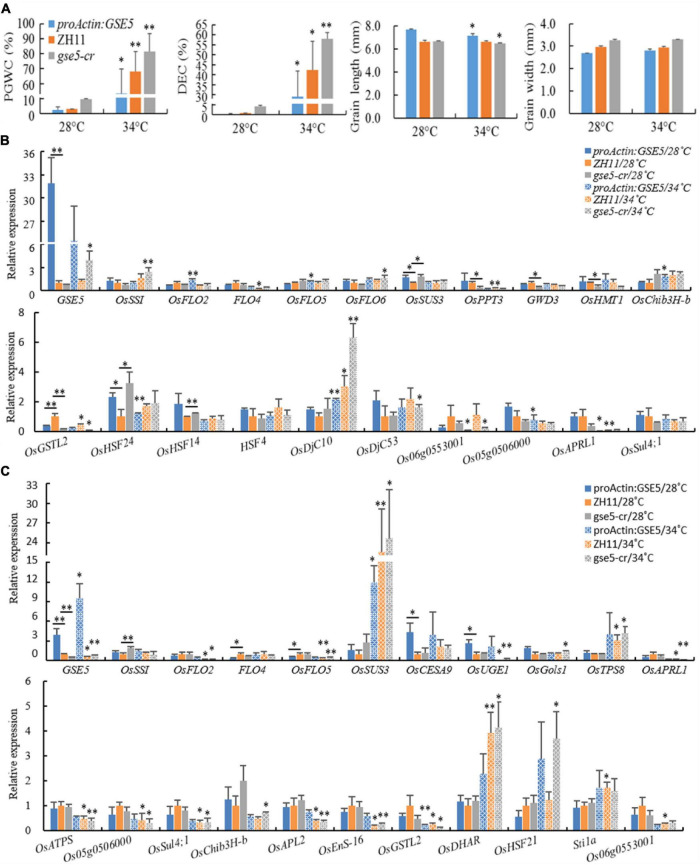
The relative expression levels of some DEGs under heat shock treatment. **(A)** The chalkiness and grain shape phenotypes of ZH11, *proActin:GSE5* and *gse5-cr* at 28 and 34°C. * Phenotype differences in a line at different temperatures (*P < 0.05 and ^**^P < 0.01). **(B)** Some genes showed different expression levels at 6 DAP under heat shock treatment. **(C)** Some genes showed different expression levels at 12 DAP under heat shock treatment. * with underlining indicates different expression levels between two adjacent lines, * without underlining indicates the different expression levels in a line at different temperatures (*P < 0.05 and ^**^P < 0.01).

High temperature during the grain-filling stage promotes rice chalkiness formation. To elucidate the expression dynamics of some genes related to chalkiness under high temperatures, a heat shock experiment was carried out in this study. ZH11, *proActin:GSE5* and *gse5-cr* were cultivated in illumination incubators with 28/26°C (day/night) and 34/30°C (day/night) at the beginning and 3 DAP, respectively. The results showed that the PGWC and DEC of the three lines at 34/30°C all significantly or extremely significantly increased, and the GL of *proActin:GSE5* and *gse5-cr* at 34°C significantly decreased, while the GL of ZH11 and the GW of the three lines showed no difference at the two temperatures (Figure 11A).

The relative expression levels of 46 genes showing different expression levels in this study or known genes associated with chalkiness were tested in dehulled developing caryopsis at 6 and 12 DAP (Supplementary Table 8). The results showed the expression of *GSE5* increased markedly at 6 and 12 DAP in *proActin:GSE5* and decreased markedly at 12 DAP in *gse5-cr* at 28/26°C compared with ZH11 (Figures 11B,C). At 6 DAP, *OsHSF24* was highly expressed, while *OsGSTL2* showed low expression in both *proActin:GSE5* and *gse5-cr*. *OsSUS3* and *OsHSF14* showed higher expression levels, and *OsPPT3*, *GWD3*, and *OsHMT1* showed lower levels in *gse5-cr* (Figure 11B). At 12 DAP, *OsCESA9* and *OsUGE1* showed higher expression levels, and *FLO4* and *OsFLO5* showed lower levels in *proActin:GSE5* (Figure 11B). *OsSSI* showed higher expression levels in *gse5-cr* (Figure 11B). This study further confirmed that *GSE5* probably regulated these genes, including *OsHSF24*, *OsHSF14*, *OsPPT3, GWD3*, *OsHMT1*, *OsCESA9*, *OsUGE1*, *OsFLO5*, and *OsSSI*, to affect the formation of rice chalkiness.

Under 34/30°C heat shock, the expression levels of *OsFLO2*, *OsFLO5*, *OsChib3H-b* (*class III chitinase homolog*), and *OsDjC10* (*DnaJ domain protein C10*, a heat shock protein) were increased, while those of *OsHSF24*, Os06g0553001, Os05g0506000, and *OsAPRL1* decreased in *proActin:GSE5* at 6 DAP (Figure 11B). The expression of *OsDjC10* was increased, while the levels of *FLO4*, *OsPPT3*, *OsGSTL2*, and *OsAPRL1* were decreased in ZH11 (Figure 11B). The expression levels of *GSE5*, *OsSSI*, *OsFLO6*, *OsDjC10*, and *OsDjC53* were increased, while those of *OsGSTL2* and Os06g0553001 were decreased in *gse5-cr* (Figure 11B).

Under 34/30°C heat shock, the expression levels of *GSE5* and *OsSUS3* were increased, and that of *OsGSTL2* were decreased in *proActin:GSE5* at 12 DAP (Figure 11C). The expression levels of *OsSUS3*, *OsTPS8*, *OsDHAR*, and *Stila* were increased, while those of *GSE5*, *OsFLO2*, *OsFLO5*, Os*UGE1*, *OsAPRL1*, *OsATPS*, Os05g0506000, *OsSul4;1*, *OsAPL2*, *OsEnS-16*, *OsGSTL2*, and Os06g0553001 were decreased in ZH11 (Figure 11C). The expression levels of *GSE5*, *OsSUS3*, *OsGols1*, *OsTPS8*, *OsDHAR*, and *OsHSF21* were increased, while those of *OsFLO2*, *OsFLO5*, Os*UGE1*, *OsAPRL1*, *OsATPS*, Os05g0506000, *OsSul4;1*, *OsChib3H-b*, *OsAPL2*, *OsEnS-16*, and *OsGSTL2* were decreased in *gse5-cr* (Figure 11C).

This study showed that the chalkiness of the three lines increased significantly under heat shock at 34/30°C, and even the floury endosperm appeared, and the GL of *proActin:GSE5* and *gse5-cr* decreased, but the GW did not. Among the 46 genes, more than 20 were differentially expressed in the three different haplotypes of *GSE5*, and the response direction of the same gene to heat shock differed among haplotypes. Under 34/30°C heat shock, *OsDjC10* (at 6 DAP) and *OsSUS3* (at 12 DAP) were upregulated, and *OsGSTL2* (at 12 DAP) was downregulated in all three haplotypes of *GSE5*.

### Improvement of Rice Appearance Quality of Zhenshan 97B by Marker-Assisted Selection

**FIGURE 12:**
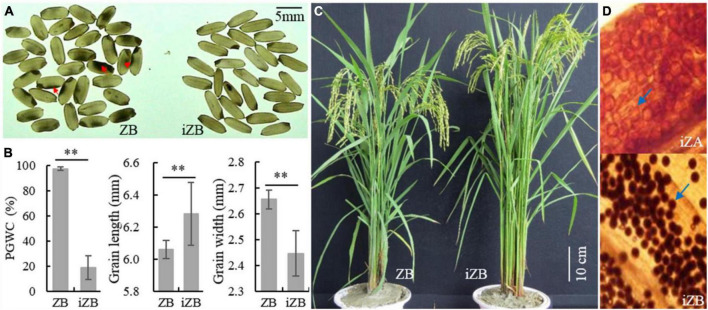
The phenotypes of ZB, iZB, and iZA. **(A)** Brown rice of ZB and iZB. The red arrows indicate chalkiness. **(B)** PGWC, GL, and GW traits of ZB and iZB. ^**^ denotes a difference at a very significant level. **(C)** Plant morphology of ZB and iZB. **(D)** Pollen granules dyed by 1% I_2_/KI. The blue arrows indicate pollen granules. The pollen granules dyed black are fertile; otherwise, they are sterile. The magnification is 10 × 10.

To improve the rice appearance quality of ZB and verify the effectiveness of *GSE5*/*qDEC5* for improving chalkiness traits in MAS, the hybridization was carried out between ZB (the recurrent parent) and ZJB (the donor of the elite appearance quality). The target interval (RM598-RM169-RM289 on chromosome 5, which is tightly linked to *qDEC5*), TIS, and GBS were carried out in the segregation population by molecular markers. The selected lines were used for further backcrossing with ZB. In BC_3_F_2_, 68 lines were obtained by TIS. According to outlier screening ($\bar{x}\pm 3\,\sigma$), 52 selected lines with a chalkiness rate less than 46.54% were obtained, with conformity of 76.47%. The PGWC of this selected population was 18.88 ± 1.29%, which was 78.72% lower than that of ZB (Figures 12A,B). In addition, the GL increased, the GW decreased, and the grain shape was slender (Figure 12B). After background genome identification and comprehensive character screening in the field, eight improved ZBs (iZBs) were selected, with average genetic background homozygosity of 99.60% from ZB (Figure 12C). Hybridization was carried out between Zhenshan 97A (ZA) and iZB, and the pollen fertility of the progenies was examined by microscopy. The percentages of sterile pollens among 21 of 50 lines were greater than 99.9%, and that of one line reached 100% (Figure 12D). Subsequently, iZB was used for backcrossing with the selected sterile lines until BC_6_F_1_ was obtained. Finally, the improved ZA (iZA) and iZB were successfully bred. This study indicated that the locus *GSE5*/*qDEC5* was an important locus affecting rice appearance quality, with a significant effect in MAS breeding.

## Discussion

### *GSE5* Regulates Chalkiness and Grain Shape

*GW5*/*qSW5*/*GSE5*, which regulates rice GW, was cloned in 2008 (Shomura et al., 2008; Weng et al., 2008), while some QTL analyses and GWASs found that some major QTLs for rice chalkiness were close to *GW5*/*qSW5*/*GSE5* (Wang et al., 2011; Gao et al., 2014; Qiu et al., 2015, 2017; Ayaad et al., 2021). *Chalk5* for rice chalkiness (Li et al., 2014) and *GS5* for rice grain shape (Li et al., 2011), which are cloned genes, locate on chromosome 5 and are also very close to *GSE5*. *Chalk5* and *GS5* are 2.03 and 1.92 Mb from *GSE5*, respectively. The sequence analysis of these two genes showed no difference between the two parental lines (ZJB and ZB) in this study, indicating that the difference in chalkiness between ZB and ZJB was not affected by these two genes (Table 2). Our map-based cloning, sequence alignment, and qRT-PCR analysis showed that *GSE5* was the candidate gene of *qDEC5*. Phenotyping of *proActin:GSE5* transgenic plants and the *gse5-cr* mutant and haplotype analysis of 69 rice varieties from around the world further confirmed that *qDEC5/GSE5* was pleiotropic because it could regulate both chalkiness and grain shape.

### *GSE5* Regulates Some Genes Associated With Carbohydrate or Protein Metabolism to Affect Rice Chalkiness Formation

In previous omics analysis, the genes or proteins involved in carbohydrate or protein metabolism played an important role in the formation of rice chalkiness (Liu et al., 2010; Lin et al., 2014, 2017a,b). In our omics analysis, the highest number (27) of DEGs belonging to carbohydrate metabolism were detected, which also showed those genes related to carbohydrate metabolism occupied an important position and role in the development of chalkiness. These genes were directly involved in starch synthesis and hydrolysis, cell wall biogenesis, glycolysis, pentose phosphate pathway, monosaccharide and oligosaccharide synthesis, gluconeogenesis, and the TCA cycle (Figure 9A). Twelve DEGs associated with protein synthesis, assembly, and degradation were found (Figure 9B). Among these DEGs, some genes have been identified to be involved in chalkiness formation in previous studies, and some have functions similar to those genes associated with chalkiness.

*WB1* and *GIFI*, which encode cell-wall invertases, regulate rice endosperm development, and loss of their function can lead to the white-belly endosperm phenotype (Wang et al., 2008, 2018). In this study, we found that a catalytic subunit of cellulose synthase gene, *OsCESA9*, involved in the formation of the cell wall was downregulated in low-chalkiness lines at 15 DAP. *OsWx*, *OsSSI*, and *OsSSIa*/*OsFLO5* are considered to be the important enzymes for starch synthesis during endosperm development (Fujita et al., 2006; Zhang et al., 2011, 2012). In this study, *OsWx* (12 DAP) and *OsSSI* (6, 12, 18, and 24 DAP) showed downregulation in low-chalkiness lines, while *OsSSIIa*/*OsFLO5* showed upregulation (10 DAP). *FLO4*, a chloroplastic pyruvate orthophosphate dikinase modulating carbon flow for starch and lipid biosynthesis during grain filling (Kang et al., 2005), was found to have lower expression in low-chalkiness lines than high-chalkiness lines by both RNA-Seq (at 15 DAP) and qRT-PCR (at 10 DAP). *OsFLO6*, which encodes a CBM48 domain-containing protein involved in compound starch granule formation and starch synthesis in the endosperm (Cheng et al., 2014), was found to have lower expression in low-chalkiness lines in both proteomics and qRT-PCR analyses. Some ER molecular chaperones, such as *OsPDIL1-1* (Han et al., 2012) and *OsBiP1/OsBIP3* (Yasuda et al., 2009), may be responsible for the occurrence of the floury endosperm. In this study, *OsBiP1*, *OsBiP2*, and *OsPDIL2-3* were found to be downregulated in low-chalkiness lines. These three genes were also found in an iTRAQ analysis but showed downregulation in the chalky tissue (Lin et al., 2014). Low expression of *FLO11/OsHsp70CP2* would lead to the occurrence of chalkiness (Tabassum et al., 2020). We found that *OsHSP70* was downregulated in low-chalkiness lines in the proteomics analysis.

In previous studies, the functional absence of most cloned genes associated with chalkiness, including *WB1*, Os*FLO2*, *FLO4*, Os*FLO6*, Os*FLO13*, and *OsPDIL1-1*, would result in the floury endosperm (Kang et al., 2005; She et al., 2010; Han et al., 2012; Cheng et al., 2014; Hu et al., 2018; Wang et al., 2018). Nevertheless, in this study, many DEGs, such as *OsCESA9*, *FLO4*, *OsFLO6*, *OsPDIL1-1*, *OsBiP1*, *OsBiP2*, *OsPDIL2-3*, and *OsHSP70*, showed downregulation in low-chalkiness lines. The low-chalkiness lines showed probably higher amino sugar and nucleotide sugar metabolism at 10 DAP, lower carbohydrate metabolism at 15 DAP, and lower protein metabolism. Therefore, the attainment of low-chalkiness or non-chalky rice is likely to be achieved not simply through the high or low expression of a few genes but the co-expression and interaction of a group of related genes, although more evidence is needed in a future study.

### High Yield and Good Quality Can Be Harmoniously Achieved in Rice Breeding

A large body of evidence has shown that PGWC is significantly negatively correlated with GL but positively correlated with GW (Li et al., 2000; Xu et al., 2004; Zou et al., 2009) and that grain thickness is positively correlated with the PGWC and DEC (Yoshida et al., 2002; Gong et al., 2008). This study showed that although *qDEC5* reduced chalkiness, it also led to an increased GL and decreased GW. Previous studies also showed that *GW2* resulted in increased chalkiness (Song et al., 2007) and *GL7* led to decreased chalkiness (Wang et al., 2015). Hence, the increase in the GW would readily lead to increasing chalkiness, thereby reducing rice quality. Conversely, an increase in GL would not increase chalkiness. This result provides a theoretical basis for long-grain and high-quality rice breeding as follows: the GW must be properly controlled/decreased to ensure low chalkiness for the sake of high quality, and with that condition fulfilled, an increased GL is desired to increase thousand-grain-weight for a high grain yield. Accordingly, both high yield and high quality can be harmoniously achieved in rice production. *qDEC5* is a very good case.

## Data Availability Statement

The original contributions presented in the study are publicly available. This data can be found here: National Center for Biotechnology Information (NCBI) BioProject database under accession number PRJNA751381.

## Author Contributions

LJ, YH, HW, YT, and JsZ conceived and designed the experiments. LJ, HZ, XJ, JpZ, RH, FL, YD, QL, and HW performed the experiments. LJ, HZ, XJ, and QL analyzed the data. LJ and HZ wrote the manuscript and other authors revised the manuscript. All authors read and approved the manuscript.

## Conflict of Interest

The authors declare that the research was conducted in the absence of any commercial or financial relationships that could be construed as a potential conflict of interest.

## Publisher’s Note

All claims expressed in this article are solely those of the authors and do not necessarily represent those of their affiliated organizations, or those of the publisher, the editors and the reviewers. Any product that may be evaluated in this article, or claim that may be made by its manufacturer, is not guaranteed or endorsed by the publisher.

## Footnotes

1
http://www.graininstru.com/productInfo_19.html

2
http://www.gramene.org/

3
http://www.premierbiosoft.com/index.html

4
http://ibi.zju.edu.cn/software/

5
http://plants.ensembl.org/Oryza_sativa/Info/Index

6
http://string-db.org/

## Abbreviations

DAP: day after pollination
DEC: the degree of endosperm chalkiness
DEGs: differentially expressed genes
DEPs: differentially expressed proteins
GBS: genetic background selection
GL: grain length
GO: Gene Ontology
GW: grain width
iZA: the improved Zhenshan 97A
iZB: the improved Zhenshan 97B
KEGG: Kyoto Encyclopedia of Genes and Genomes
MAS: molecular marker-assisted selection
NIP: Nipponbare
PGWC: the percentage of grains with chalkiness
PPI: protein-protein interactions
SEM: scanning electron microscopy
TIS: target interval selection
ZA: Zhenshan 97A
ZB: Zhenshan 97B
ZH11: Zhonghua 11
ZJB: Zhenjia B.
