# Supplementary material for: Identification and Pleiotropic Effect Analysis of GSE5 on Rice Chalkiness and Grain Shape
Source: Front Plant Sci. 2022 Jan 20;12:814928. doi: 10.3389/fpls.2021.814928 (PMC8810533; doi:10.3389/fpls.2021.814928)
Supplement: Supplementary file 1 [file Data_Sheet_1.doc]

**Identification and Pleiotropic Effect Analysis of *GSE5* on Rice Chalkiness and Grain Shape**

**Supplementary table 1 | SSR primers used for** genetic background selection.

| No.of primers | Name of SSR markers | | | | | | | | | |
| --- | --- | --- | --- | --- | --- | --- | --- | --- | --- | --- |
| 95 | RM3 | RM5 | RM9 | RM14 | RM16 | RM17 | RM23 | RM70 | RM71 | RM80 |
|  | RM86 | RM101 | RM102 | RM105 | RM109 | RM110 | RM111 | RM154 | RM168 | RM186 |
|  | RM202 | RM204 | RM205 | RM207 | RM208 | RM209 | RM211 | RM214 | RM215 | RM222 |
|  | RM227 | RM228 | RM229 | RM233 | RM241 | RM252 | RM254 | RM257 | RM258 | RM261 |
|  | RM264 | RM271 | RM273 | RM279 | RM280 | RM282 | RM283 | RM286 | RM290 | RM295 |
|  | RM304 | RM311 | RM313 | RM314 | RM316 | RM324 | RM333 | RM335 | RM337 | RM339 |
|  | RM401 | RM410 | RM424 | RM428 | RM434 | RM440 | RM442 | RM445 | RM447 | RM457 |
|  | RM462 | RM463 | RM471 | RM480 | RM485 | RM488 | RM492 | RM493 | RM496 | RM500 |
|  | RM503 | RM508 | RM511 | RM514 | RM524 | RM526 | RM530 | RM543 | RM549 | RM563 |
|  | RM570 | RM579 | RM581 | RM588 | RM589 |  |  |  |  |  |

**Supplementary table 2 | The haplotypes and phenotypes of 69 rice varieties from around the world.**

| **Haplotypes** | **Varieties** | **GL of milled rice(mm)** | **GW of milled rice(mm)** | **PGWC (%)** | **DEC (%)** | **Country or region** |
| --- | --- | --- | --- | --- | --- | --- |
| *GSE5^ZJB^* | Notai | 5.8 | 2.4 | 48.5 | 10.8 | America |
|  | XIANG SI MIAO 2 | 5.5 | 1.9 | 22.5 | 6.3 | China |
|  | IRAT 134 | 6.1 | 2.7 | 92.5 | 51.3 | Côte d'Ivoire |
|  | HIJOLEE | 5.7 | 2.6 | 51.5 | 17.3 | Bangladesh |
|  | IRAT 3178 | 6.6 | 2.3 | 94.0 | 44.1 | Côte d'Ivoire |
|  | Wen Sheng 4 Hao | 5.8 | 2.3 | 95.5 | 44.9 | China |
|  | Gu Mei 3 Hao | 6.6 | 2.6 | 96.5 | 37.3 | China |
|  | Cheng ai 29 | 6.1 | 2.3 | 81.5 | 22.2 | China |
|  | IR2307-27-2-2-3 | 6.9 | 2.1 | 28.5 | 8.5 | IRRI |
|  | TM 10265 | 5.4 | 2.3 | 58.5 | 13.6 | India |
|  | Ma Xiang Zhan | 5.5 | 2.0 | 33.0 | 8.2 | China |
|  | SELECCION 46-113 | 7.0 | 2.2 | 78.0 | 21.5 | Panama |
|  | ECIA 89-1-3 | 7.2 | 2.3 | 66.5 | 19.0 | Cuba |
|  | ECIA 157-S8-1 | 6.8 | 2.2 | 26.5 | 5.6 | Cuba |
|  | A1454 | 6.6 | 2.0 | 47.5 | 15.3 | IRRI |
|  | IR8608-65-2-3 | 6.6 | 2.0 | 37.5 | 11.7 | IRRI |
|  | IR-BB8 | 6.8 | 2.1 | 19.5 | 5.6 | IRRI |
|  | PAU 41-281-1-1 | 6.4 | 2.1 | 44.0 | 14.9 | India |
|  | BG 1165-2 | 6.4 | 2.0 | 54.0 | 19.2 | Sri Lanka |
|  | C.MEDIO 19 | 7.1 | 2.2 | 12.5 | 4.6 | Cuba |
|  | GUI HONG ZHAN | 5.8 | 1.8 | 0.0 | 0.0 | China |
|  | 91499 | 7.3 | 2.3 | 12.5 | 2.3 | China |
|  | PERARK | 6.8 | 2.0 | 2.5 | 0.4 | America |
|  | C.CORTO 2 | 7.9 | 1.9 | 12.5 | 2.8 | Cuba |
|  | C.CORTO 13 | 7.1 | 2.1 | 9.0 | 2.2 | Cuba |
|  | C.CORTO 21 | 6.9 | 2.1 | 7.5 | 2.3 | Cuba |
|  | C.MEDIO 16 | 7.9 | 1.9 | 23.5 | 6.6 | Cuba |
|  | ARTIGLID | 8.0 | 1.9 | 17.0 | 6.4 | IRRI |
|  | PIN 24 | 5.3 | 1.6 | 2.0 | 0.3 | India |
|  | IRI 358 | 7.2 | 2.0 | 27.0 | 8.4 | Korea |
|  | Hua Zhan | 6.1 | 2.0 | 16.0 | 4.4 | China |
|  | Min Hui3301 | 7.3 | 2.1 | 66.0 | 26.3 | China |
|  | Jiafuzhan | 7.3 | 2.0 | 0.8 | 0.2 | China |
|  | Xiang Ya Xiang Zha | 7.4 | 1.6 | 1.0 | 0.4 | China |
|  | Ming Hui 63 | 6.5 | 2.2 | 70.0 | 22.6 | China |
|  | Shu Hui 498 | 7.0 | 2.3 | 11.0 | 3.2 | China |
|  | Huang Hua Zhan | 7.2 | 1.6 | 0.0 | 0.0 | China |
|  | Shu Hui 527 | 7.5 | 2.3 | 21.0 | 4.9 | China |
|  | Zhenjia B | 6.6 | 2.1 | 1.0 | 0.17 | China |
| *GSE5^ZB^* | GZ 2175-5-6 | 5.2 | 2.5 | 73.0 | 15.0 | Egypt |
|  | Ke Te | 6.7 | 2.6 | 100.0 | 72.9 | Côte d'Ivoire |
|  | Lung Sheng 1 | 5.6 | 3.1 | 96.5 | 44.9 | Côte d'Ivoire |
|  | PTB18 | 5.4 | 2.7 | 100.0 | 79.8 | India |
|  | Gan Zhao Xian 25 Hao | 5.5 | 2.8 | 100.0 | 62.7 | China |
|  | 30014 | 5.6 | 2.7 | 100.0 | 46.1 | China |
|  | BN88004 | 6.2 | 2.6 | 100.0 | 46.0 | China |
|  | Da Gu Ai 3 Hao | 5.6 | 2.6 | 100.0 | 48.6 | China |
|  | 800456 | 5.7 | 2.7 | 100.0 | 57.0 | China |
|  | 39-21 | 5.7 | 2.5 | 100.0 | 57.1 | China |
|  | Gui Fu 3 Hao | 5.7 | 2.4 | 100.0 | 64.1 | China |
|  | Jin Xuan 2 Hao | 5.4 | 2.5 | 100.0 | 62.0 | China |
|  | CNM8 | 5.6 | 2.4 | 72.0 | 21.2 | India |
|  | IR8608-75-3-1 | 6.0 | 2.0 | 61.5 | 17.7 | IRRI |
|  | Guang Chang13 | 5.8 | 2.5 | 98.0 | 30.8 | China |
|  | Ai Jiao Nan Te | 5.6 | 2.5 | 62.0 | 19.9 | China |
|  | Zhenshan 97B | 5.6 | 2.6 | 77.0 | 14.1 | China |
| *GSE5^NIP^* | Kitaake | 5.2 | 2.9 | 16.0 | 3.0 | Japan |
|  | Tai Nong 71 Hao | 5.1 | 2.9 | 34.0 | 10.8 | China |
|  | Yan Feng 47 | 4.9 | 2.8 | 36.0 | 20.2 | China |
|  | Feng Jin | 4.9 | 2.7 | 6.0 | 2.8 | China |
|  | Dong Dao 4Hao | 5.2 | 2.9 | 35.0 | 15.5 | China |
|  | Fujisaka 5 | 4.8 | 2.8 | 22.0 | 7.1 | Japan |
|  | Long Geng Xiang 1 Hao | 5.1 | 2.7 | 12.0 | 3.6 | China |
|  | Jia Hua 1 Hao | 5.0 | 2.6 | 14.0 | 7.2 | China |
|  | Koshihikari | 4.9 | 2.7 | 19.0 | 6.5 | Japan |
|  | Shen Nong 606 | 4.8 | 2.6 | 7.0 | 3.0 | China |
|  | Nipponbare | 5.1 | 2.7 | 3.0 | 0.6 | Japan |
|  | SHINKWANG | 6.3 | 2.4 | 63.5 | 19.5 | Korea |
|  | Zhong Hua 11 | 4.2 | 2.4 | 3.5 | 0.8 | China |

**Supplementary table 3 | SSR primers on chromosome 5 used for polymorphism analysis between ZJB and ZB.**

| No.of primers | Name of SSR markers | | | | | | | | | | | | | | | | | |
| --- | --- | --- | --- | --- | --- | --- | --- | --- | --- | --- | --- | --- | --- | --- | --- | --- | --- | --- |
| 59 | RM13 | RM26 | | RM31 | | RM39 | | RM87 | | RM122 | | RM146 | | RM153 | | RM159 | | RM161 |
|  | RM163 | RM164 | | RM169 | | RM173 | | RM178 | | RM188 | | RM194 | | RM233B | | RM249 | | RM267 |
|  | RM274 | RM289 | | RM291 | | RM305 | | RM334 | | RM405 | | RM413 | | RM421 | | RM430 | | RM437 |
|  | RM440 | RM459 | | RM465C | | RM473B | | RM480 | | RM507 | | RM509 | | RM516 | | RM534 | | RM538 |
|  | RM548 | RM574 | | RM592 | | RM593 | | RM598 | | RM18132 | | RM18136 | | RM18165 | | RM18236 | | RM18291 |
|  | RM18379 | | RM7118 | | RM5994 | | RM3683 | | RM7293 | | [RM2848](http://www.gramene.org/Oryza_sativa/Location/Marker?m=RM2848;r=5:7793291-7793483) | | RM7349 | | RM5874 | | RM3328 | |
|  | RM511 | RM512 | | RM519 | | RM558A | | RM28099 | | RM28208 | |  | |  | |  | |  |

**Supplementary table 4 | ‘XM’ SSR primers newly designed and synthesized on chromosome 5.**

| Name of primers | Forward Primer | Reverse Primer |
| --- | --- | --- |
| XM1 | GGTTTGAGTTTGGTTCTTGGG | CCTCTTCTCTTCGGCTGCTT |
| XM2 | GTCACTTGCCTCCTCCAAT | ACGAGAGCGAGAACGAACA |
| XM5 | GGCTTCTTCCTCCTCTTGA | CAGGTTTACTCACTTGCCTC |
| XM3 | TTCTGCTTTGTGCTGCCGAG | TCCTGTGGCTGGTGCTTGTA |
| XM4 | GATTGTGGAGTGTTGGAGTG | CAGGTGAGTAGGTTCTTGTTG |
| XM6 | CTGATGGAGAGAGAGACGA | GGTTCTTTGACAGGTTGAC |
| XM7 | GACAGGCTCCGACATAGTTA | ACGCCATTCTCACCCAGAT |
| XM8 | CATCACACATCTCTCCACT | GAAGAAGCCGAGCAAGAA |
| XM9 | TTCCGCCCGCACAAATCAT | GCTGTATGTTGCTCAGGCT |
| XM10 | GCCTTTGACAGTTTGACCC | AAGTGGAAGTGGACGGACT |
| XM11 | TGGAATGGTTACTGACAGGGC | GCTTGCTGCCTCCTTCTTG |
| XM12 | AGGTGGTAAAGTGTGTGC | AGATGGAAACACGAAGCG |
| XM13 | TTCAGGCTCGTGGAGGAG | CCCAGCAGGAGAAACAAAGAA |
| XM14 | CGCTACACCAATCCCTACCCA | GGTTGCTGCTGTGGGACG |
| XM15 | TGGGCAAGCGTGGATTCTCT | TGGTGCTCTTGCCTTTGCCA |
| XM16 | GAAGCGAAGCGAAGCGAAGA | GTAGACCTGCTCGGAGGAGT |
| XM17 | TCCCTAACACCACTTGGC | ACTCTCACCCACCTCCAC |

**Supplementary table 5 | SSR primers used for fine mapping of *qDEC5*.**

| Name of  primers | Forward Primer | Reverse Primer | Position on chrom. |
| --- | --- | --- | --- |
| RM3419 | TGCTGCTATTCCTCAAGACAAACC | CTTGGTGAAACAGTGCTCTCTGG | 5228602 |
| RM1089 | CAGAAGGATTATCTCGATACC | AATAGGGCTTGAAATAAATTG | 5299836 |
| RM18039 | ACGATTGACCACCATGAGCTACC | ACCGGGTGGTGTGAACTTATTCC | 5446668 |
| RM3917 | CGAACGAACGAGGACGAACG | TCTAGGGCTAGCTCTTCCACTCAGG | 5585026 |
| RM18057 | CTTGTGCCCAACAAGAGGAGAGC | GGATGCTCACCGGGATAGATGC | 5858378 |
| RM18060 | AGCAGCGGCGACCTCTTCTACG | CGGACGGACTTCCTGTTCTTGC | 5883930 |
| RM18076 | ACATGTCCCTGCCCGTAAACC | GTCCACTCCAGCTCCTGCTTCC | 6251194 |
| RM18084 | TCTATCGTACGTATTGACCAGTCC | TACCCTGCACAACACTATCTAGC | 6373564 |
| RM18096 | TACACATAGGATACCCATACCC | CAATCTTTAGTTCCGGTTGG | 6549932 |
| RM4691 | CCATCAAGAGATAGTGCTCCAACC | ACACAGCCTATGTTTAGGGTTTCC | 6941334 |
| RM18120 | TCCAAACACCACCTGAAACTGC | TGCTTTCTGGTAATCTCGTCTCG | 7193979 |
| RM18142 | ACGTGTGTAGGGCATCTTCTCG | GGCTCTGAGCTACCCTTACATGG | 7705313 |
| RM18153 | CGTACGACCTATCCCTATGAGACG | TCCGGATCACATTTCTGGTTAGC | 7841032 |
| RM1366 | CCTCCTCAGGGCCAAACATTGC | TTGAGTGGCTCTGGACCTTCTTCAGC | 2855577 |
| RM17908 | CCGGATAAGCATCCTCCTCACC | AAAGCTGCCTCCTCGTTGTTGG | 2917186 |
| RM17911 | ATTGTGAAACGGAGGAAGTAGG | CCTCTCCGTTCTCCCTAACG | 2978109 |
| RM17914 | CCTACCACCACCAGCTTCTTGC | ATGAGCACTGAGCAGAGGATGC | 2990615 |
| RM17931 | CAGCAAGTCCCTAACACTGAAGAACC | CCCTCATCACCATCTCCAACC | 3167336 |
| RM17940 | CAATTGTTTGCCTTGGTTGTGC | CAGAGCTCCTGCCGTTGACC | 3331603 |
| RM17950 | GGAAATGTGCATAGGTAGTTCAGG | GAGTTGGGAACTGCTACAAACG | 3490890 |
| RM17958 | GCCAAACTGATCTCTGCAAGC | GCACGTCACATCCTAATCAACG | 3746110 |
| RM17981 | GGTTTGACCCATGGGAGAGG | AACTGCACACGTATGGACTTGG | 4434595 |
| RM17990 | TCTCCACACAATACAAGTCACG | GAGAGTTGGAGAGAAAGGAAGG | 4627860 |
| RM18004 | CTCGAAGCTATTAGCCGGGATCG | ATCTTCTTCCTCGCCGTCTTCC | 4973068 |
| RM18055 | AGATCTCCTCTCAGAGTCTACCG | CACTGAGTATAATCCCTGCAACC | 5796052 |
| RM18068 | CAGCACTGACCACACTGTAATGG | GAGTTTGGCTAGAACACATCATGG | 6051033 |
| RM18092 | CTCCTTGGGTGAATCCTATGTGC | GAAACCGACTCAAATACGATGACG | 6500052 |
| RM18107 | CGTATGGACTTGCCTTGAGTCG | TCCAATCTGCCAAGCTTTACACC | 6742128 |
| RM2998 | TCTAGATACACCGTCTCAATGG | AAAGAGGTAGCTAGGTTGAAGG | 7150315 |
| RM17988 | CAGGCTGCAACCTCAAATGG | CTCCTCCTCCTCCTCGTCATCC | 4625052 |
| RM18001 | TGAGCGAGCTGTAAGTTCCTTTCC | CGATCGTAAGCTCCCTTCTTTGC | 4912609 |
| RM18011 | TATTTGCATGGCCCTGTTTGC | CAATTCATAACGTCGGTCCTTCC | 5093275 |
| RM18018 | AAATTATCTCCCTCCCTCTCTCC | CCAGATTCAACATTCAGACACC | 5173094 |
| RM18019 | CGTGGTTCTAGAGATGAGAAAGAGG | GGTACTACAGTCCCAGAAGAAAGTGG | 5204319 |
| RM18032 | AGCACCTGACCTGACCTGACTCC | GACCTGACCGGCTTAAACTATCTCC | 5248676 |
| RM18040 | CCGGGACTAAAGATAGAGCTGACC | AACACAATTCACCGGTCCTTAGC | 5479016 |
| RM18047 | GTTTCTGTCCAAGGGAAATCAGC | AGGACCATTAACCTGAGGATTGG | 5669106 |
| RM18048 | GATCACTAGCAGAGGCACAAGAGG | ATGGCAAGTTAACAGCGACATCC | 5715624 |
| RM18053 | GAGACCAGAGGGAGACAAAGAGAGG | CTTAGGTCTCCCGACAGTCACG | 5771847 |
| RM18057 | CTTGTGCCCAACAAGAGGAGAGC | GGATGCTCACCGGGATAGATGC | 5858378 |
| RM18060 | AGCAGCGGCGACCTCTTCTACG | CGGACGGACTTCCTGTTCTTGC | 5883930 |
| RM18063 | CCGAACAGATGAGGAGCAGAGG | TCGGAAGACATCTGTCGGTAAGACC | 5935239 |
| RM18070 | CTTTCGCCTCCTGCATTTGG | AAGGGAAAGCCCAATACTGTTCC | 6097462 |
| RM17907 | CTCCTGCTCAACCAAACCAAACC | CAATATCCATGGCCGATTCAGG | 2912125 |
| RM17909 | TCTCACCAGTATATGCTCTTCG | AGAAGTCGAGGGTAAGAAATCG | 2917862 |
| RM17923 | CATGGCCAGTGCAAATTCAAGC | CACATTCCTCTCCACTTTCTTGTCG | 3068460 |
| RM17935 | CTAAAGGCCTAGCAATATCTCC | CACTCAACTTTCCCTATTAGCC | 3227675 |
| RM17947 | GTTCTTGCCTTCTCGGCATGTGG | GGTCACTGATTCCACCATTCACACC | 3442245 |
| RM17954 | ATTTCAGTACAAGGCACCCATGC | GTAGACGAGGGAGTACCAACTTGC | 3591046 |
| RM17966 | AAACTGTTACATGGGCTGGTTGG | CCACTCGGTGTCAACATTAATACG | 3931431 |
| RM17971 | TGATCTCACCTCTTCCCTACAAGC | GCTCCCAAATCTTGGTCTTGC | 4113575 |
| RM17976 | CGGTCCGTTCTTGCCTTCTTGC | ACGCCTCCTGCAACGAACACC | 4203747 |
| RM17984 | TGATCAAACACCAACGCTAGTCTCC | GGTTTCGGGTGGTTGCTTACC | 4500224 |
| RM17989 | ACGAGGAGGAGGAGGATGACG | GGCTCGACTTCGAGGACACC | 4625198 |
| RM17998 | GAACACTAGGCGCATCCATTCC | ATTAGGGAGCGTTGGATTGTTTCC | 4868616 |
| RM18007 | AGCGAACGAATGAGGACGAACG | ACGGACGACTCCGACAACACG | 5036853 |
| RM18035 | CCGTGGTGATAAACTGTTAACTGAGG | CGCATATGCATCAGCATCACG | 5305663 |
| RM18039 | ACGATTGACCACCATGAGCTACC | ACCGGGTGGTGTGAACTTATTCC | 5446668 |
| RM18041 | TAGGGCTAGCTCTTCCACTCAGG | AACGTGAACGAGAACGAGAACG | 5483775 |
| RM17980 | TTGCATCGGCATATAAGGATCG | AAGCACTGATCCGAAGGTAAAGC | 4433704 |
| RM17982 | GTCTGTTGCGCCATTTAGTTTCG | CGAGAGGAGTTCTGGCGTTCC | 4439335 |
| RM17983 | ATCAACAGGGTGCTAACCAGAGG | GCACGAGTCCCTTGCTGACC | 4465410 |
| RM17996 | CCGTCTGCTTCCACGTCTGC | TGAGGAAGGAGGTGGTGTTAGTGG | 4757414 |
| RM18038 | AGCCCGCTGTCATTCTCTCTCC | GGTGGAATCAGAGATGAACACATTGC | 5420426 |
| RM18045 | CACCGGTCCTTAGCTTCTGAGC | TTACCAACCGGGACTAAAGATCG | 5589651 |
| RM1089 | CAGAAGGATTATCTCGATACC | AATAGGGCTTGAAATAAATTG | 5299836 |

**Supplementary table 6 | ‘CH5’ series of newly designed and synthesized SSR primers.**

| Name of primers | Forward Primer | Reverse Primer | Position on chrom.5* |
| --- | --- | --- | --- |
| CH5-1 | AACGAGCGAACGAATGAGGA | TCAGGCTTGACGGACGACT | 5094554 |
| CH5-2 | CTTCACTCGTTGTAACGCACA | AGCACAAATCTTAGCAGCCATC | 5166298 |
| CH5-3 | CGGCGTCGAAGTCGTATCTA | GCTGTTTGCTGCAAGGTTGA | 5207664 |
| CH5-4 | AGCAAATTATCTCCCTCCC | GCAACAGTAGCGTAATGTCTAA | 5230795 |
| CH5-5 | AACGGCAGCAATTCCTCAA | GTTGGACATGGACTCCTCTC | 5266954 |
| CH5-6 | CTATTCCTCAAGACAAACCTCATCA | GACAGTGCTAACATTCTATCCTTCA | 5284972 |
| CH5-7 | TTTGACTCCCCAGAAGGA | GGGCTTGAAATAAATTGAAAACT | 5356206 |
| CH5-8 | GCAGGAAGAATCCAAGAATT | AGGACCATAGATGTGAGTTA | 5379417 |
| CH5-9 | TCCTCTCCTCTGAGCGTCTG | GCGGGTGCTTATGTCTTCCT | 5464605 |
| CH5-10 | CCATGCTCCATGTAGCACCAG | CTAGGTTCGTATTATCCAACTTCCA | 5526910 |
| CH5-11 | CGCTACTCCCTCCGTTTCAC | CGCTTCCTCTAGGCTCATCTTG | 5540015 |
| CH5-12 | GTCACTTGCCTCCTCCAATTCC | GTGAACGAGCGAACGAACGA | 5565509 |
| CH5-13 | GGTCTCTAAACTTTAGGTGT | GCGTCAAGTTTACACATCT | 5622977 |
| CH5-14 | ACGAGCGAACGAACGAGGA | GCTCTTCCACTCAGGCTTGAC | 5666885 |
| CH5-15 | CGAACGAATGAGGACGAACG | TCAGGCTTGACGGACGACTC | 5094515 |
| CH5-16 | ATCTCGGCTTCTCCTTCACC | TCATCTGCGGTGGCGATT | 5202686 |
| CH5-17 | CTTCGCTAGGCAGCCATCA | TAACTTTAAGCCGAGCCATCAG | 5414300 |
| CH5-18 | TAGTCGTCCACACCACACAGTC | AGGGAGAGCGATGCGAGAGA | 5481084 |
| CH5-19 | CACGGTTAATGTCTCACAACAGTAG | TGGTAGCTCATGGTGGTCAATC | 5502803 |
| CH5-20 | CCCTGTTGCTGCATGGTTTA | CGAGTACTACTACGTCGAAATCG | 5560853 |
| CH5-21 | GGCAGTCCAGTCAGCATTCT | CTCGTCGTCATCATCCTCATCC | 5592240 |
| CH5-22 | AGTGAGTACTACTACGTCGAAATC | CCCTGTTGCTGCATGGTT | 5671244 |
| CH5-23 | CAGCGTCGAGCGAATCTTC | ACCCTATCCATATCTATTCCCTTCT | 5433535 |
| CH5-24 | CGTCTTCCACCCGATGATGTT | AGGTTGCTCCTCCTCCTCAC | 5509995 |
| CH5-25 | GCGTTTGTTCGTGTCTCTCT | TTTATACCTTTGAGCCCGTGC | 5526521 |
| CH5-26 | GCTAAGTAGTGAGAGCCGAGAG | TTTCACCCTTACCGCAAATCTAG | 5549409 |
| CH5-27 | CACCCTAAATTGCAAAGATCAAAGC | CTCTCCAGAAGGATATCTCGATACC | 5587591 |
| CH5-28 | GCAGTCCAGTCAGCATTCTATT | TCGATGGAGACGACAAGCC | 5595806 |
| CH5-29 | TGTGTGCGTGAGAAAGAGAGG | GGCAGTCCAGTCAGCATTCTAT | 5596593 |
| CH5-30 | ATGATGGATCATATCACCACCTAG | CAGACCTTACGAAGAACGAGATAA | 5637363 |
| CH5-31 | ACAAGGACGGTTAGAATGAT | TGGAGGTGGTTCCTATGATAA | 5707416 |
| CH5-32 | CCCATTACAGAGGCAATCACG | CTTAAATAGGGAGTTATTGGAGGCT | 5711767 |
| CH5-33 | ATCGCCTGCCCGTTAGAGA | GAGTTATTGTAGGTTCCCTGTTGAC | 5716917 |
| CH5-34 | TTCCTGACATTGCGTGTTAGTT | CCTGAGGATTGGATTACATAAGTTG | 5750871 |
| CH5-35 | CCACGGTGTAGTTACATCCTT | CGGTGCTGCATGATCAACAT | 5754680 |
| CH5-36 | GCTGCTTCTTTGACCTTCTCC | TGCTGACATTGAACGGGCA | 5361945 |
| CH5-37 | TGAGACGACGAGGAGAGCTT | AGAACAGAAACCCACATGATTGATC | 5366715 |

**Supplementary table 7 | Primers of 20 genes related to chalkiness or starch biosynthesis for qRT-PCR.**

| Gene | Name of primers | Forward Primer | Reverse Primer | RAP ID |
| --- | --- | --- | --- | --- |
| *GSE5* | q09520 | AATCTCCCGCACCTCCACCA | GTGTCCACCTCCACGATCTTGG | Os05g0187500 |
| *OsWx* | qWx | TGTGGCTGAGATCAAGGTTGC | CTCCAGTGTCAGGTCCGTAGAT | Os06g0133000 |
| *OsSSI* | qOsSSI | TCATGGATGTGAAGGAGCAA | TGGCAGTGAACCACAAACAT | Os06g0160700 |
| *Chalk5* | qChalk5 | TGTTCATCTGCGTCTCAGCT | AGGCCGAAGATCACGTTCGTC | Os05g0156900 |
| *OsGW2* | qGW2 | ATGCCTGTAACTGAGCCATCT | CGAGCACTCTCCTTCTTCTCT | Os02g0244100 |
| *OsSSIIa* | qOsSSIIa | GATCGACCAGGATGACGATT | GGGTAAAGCACCTGCAACAT | Os06g0229800 |
| *OsSSIVb* | qSSIVb | GAGCTGCTCCTGCTCAAGAT | ACACAATTGCACCCTTGACA | Os05g0533600 |
| *OsAmy3A* | qAmy3A | CGGGCATGATAGGCTGGTAT | CGTTGATCTCTTGCTTGAGGTT | Os09g0457400 |
| *OsFLO2* | qFLO2 | CACACCCTCCAGCAATATCA | CCTTCTGCGACTGCTTTTCT | Os04g0645100 |
| *FLO4* | qFLO4 | CATGCACTGTTCGAGGAGAA | GGGAAATGGCTCTCCCTTAG | Os05g0405000 |
| *OsFLO5* | qFlO5 | GCCTGCCCTGGACTACATTG | GCAAACATATGTACACGGTTCTGG | Os08g0191433 |
| *OsFLO6* | qFLO6 | AGGCGATCTACGAGTTCATGC | ACCCAGTCCCAAATTTCGTCTC | Os03g0686900 |
| *OsEnS-51* | qGlobulin1 | ATCGAGAACGGCGAGAAGT | GGACGGAGATGGTATGGAGA | Os03g0663800 |
| *OsEnS-57* | qGlobulin2 | CGACGAGGTGTTCTACGTCA | GTGTTGGCGGAGTAGACGAT | Os03g0793700 |
| *OsISA* | qISA | CATTGCTGAAGCATGGGATGCG | GCCAGCGTAAATCCATCATGCG | Os08g0520900 |
| *G6PIb* | qG6PIb | GTTGCACGAAGCATCAAAGA | ATGTTTCGCAACAGCATCAG | Os06g0256500 |
| *OsRab5a* | qOsRab5a | ATTCCACGAGATCGGTGAGTT | TTGAGCCTGCTGTCCTTGAA | Os12g0631100 |
| *OsSUS3* | qSusy3 | CATGTACCCCCTGCTCAACT | GTCAGCTGTAATGCCTGCAA | Os07g0616800 |
| *OsGIFI* | qGIF1 | CATCGCGCAACCCGAACATG | TGTCGATCAGGCTCCTCAGAG | Os04g0413500 |
| *OsAPL2* | qOsAPL2 | CCGCTGTTCCTGTTGGAGGATG | GCAGCCAATACCTGCACAGACC | Os01g0633100 |

**Supplementary table 8 | Primers of the genes related with chalkiness or DEGs for qRT-PCR.**

| Gene name | Name of primers | Forward Primer | Reverse Primer | RAP ID |
| --- | --- | --- | --- | --- |
| *OsHSF21* | qHS546800 | GTCATCTCCTGGAACGACGACG | TCACCTTCCGGCGGTGTATCT | Os08g0546800 |
| *OsHSP90* | qHS716700 | AGTGAAGAGAAGAAGCCCAAGACA | AACATCTCCCTCAGCAGTGAAGTG | Os06g0716700 |
| *HSF4* | qHS745000 | GCCGTTCTTGTGCAAGACGTAC | GTGTTGAGCTGGCGGACGAA | Os03g0745000 |
| *OsDjC53* | qHS195800 | AAGGGCGTGAACAAGCGGTG | CGGCCTCTGCTTTCTCCTCTTG | Os06g0195800 |
| *Os11g0696600* | qHS696600 | GCTCAAGCCACGAGGGATCTTT | GGACCAAAGGGCAGGATCAACA | Os11g0696600 |
| *OsHSF24* | qHS526600 | GGGAGAAGCGTTTGTTGTGTGATAT | CCGACCCAGAGTTTGACGACAG | Os09g0526600 |
| *Sti1a* | qHS644100 | CGAGACCGCGATCCAGCATTAC | CAAGTTTGGCTAGGGCTGTTCCTT | Os02g0644100 |
| *OsHSF14* | qHS568700 | AGCACCGATGAGACCATCTCGT | CTCTGTATCCCACCGAGCAGGT | Os04g0568700 |
| *OsHSF6* | qHS419300 | CAACAGCTTCGTCGTCTGGGAC | TTGTTGTGACGGAGGTGCATTGG | Os10g0419300 |
| *OsDjB1* | qHS239100 | CCATTGAGAACCGGCTTCCAGTC | TCAGCAGGTTTCATGTTTGGAGCT | Os01g0239100 |
| *OsDjC10* | qHS606900 | TGTTGTCTGACGAGAAGAGGAGGG | ACGAGGCGAAATCCTGCATCATC | Os01g0606900 |
| *OsDjB7* | qHS562300 | GCTCTACAAAGGCACCACCAAGAA | TGCGGCTTCTCGTCGATGATGA | Os05g0562300 |
| *Os08g0464000* | qHS464000 | GGTCAATCAGCGGCGTCAATGA | CTCCGTGTTCTCCACCACAGTTG | Os08g0464000 |
| *OsSTA1* | qSM110700 | GAGACGGTGAATGATCTGCGAGAA | CATGGAAGAGCGTAACCTTGATGC | Os01g0110700 |
| *GWD3* | qSM297500 | TGACAATGCTGTCTCTACAGAACCA | CTTCTGAAAGCTCAAGAGCAGCAAA | Os12g0297500 |
| *OsChia2a* | qSM542900 | GGTAGTTCTGACCAGTTCCAGTGG | GAACCAGATGGCCGTCTTGAAGG | Os10g0542900 |
| *OsChib3H-b* | qSM700900 | CTTCTTCATCGACAACGGCCCAG | CACTTGTTCCACTGCGCCATGA | Os11g0700900 |
| *Os03g0747200* | qAPR747200 | GCCTAGTGCGTGGTTCAAGACAA | TGGAAACCCTGGAGGAGCATCC | Os03g0747200 |
| *OsHMT1* | qAPR422200 | CGACATGATGGCGGAGTTCCTG | GAAGCCCTGAATTGTGGCCTGAT | Os10g0422200 |
| *OsATPS* | qAPR743900 | ATGTCCGTGCCCATCGTCCT | TCACCAGCATTTGTAATCGCCTCA | Os03g0743900 |
| *Os05g0506000* | qAPR506000 | GACCTCTTCCATGTCGTCCACAAG | TCTGATGGCTTCTATCGCCTCCTT | Os05g0506000 |
| *OsAPRL1* | qAPR509800 | TCGCCGCTGGAGATCATGGAT | GCGTCCGGGAACATGTACTCGA | Os07g0509800 |
| *OsSul4;1* | qAPR240500 | GATGCGGACGTACAGGTTGAAGG | GACAAGAGCCACTGGACCCACT | Os09g0240500 |
| *Os02g0232050* | qHS232050 | GTGTAGAGGCCGGTGAAGTCGA | AGATGCTCGCCTTCCTCGTCAA | Os02g0232050 |
| *Os06g0716750* | qHS716750 | CAGGAAGGTTGAAGCCGCTCTC | GCGTGGTGGTGACATCCAAGTAC | Os06g0716750 |
| *Os06g0553001* | qHS553001 | ATGGTGCGCTGCTCCTGCTT | CAGGGTTTCCGCAAGGTGGATC | Os06g0553001 |
| *OsGSTL2* | qHS283100 | GCCTGCTTGGTACAAGGAGAAGG | GGCGACTATGGATGAGTACGATGC | Os03g0283100 |
| *OsMIOX* | qHS561000 | CATCGAGCTGCTCAACGAGTTCAT | GGAAGGTGTCACCGACGACTGA | Os06g0561000 |
| *OsDHAR* | qHS116100 | GCTGACTCTGGAGGAGAAGAAGGT | TCCCACTGATGCATACTCAGGAGG | Os05g0116100 |
| *OsCESA9* | qCESA9 | CGATGCTGCACGGCAAGATGA | TCGTCCATCCTCTCCTTCCAGC | Os09g0422500 |
| *OsUGE1* | qUGE1 | ACCTCATCGGCACCATCACTCT | CCTCACCAATGTACCCGCTTGG | Os05g0595100 |
| *OsGolS1* | qGolS1 | CCAAGTCCCTGCTCGACACTCT | CTGTCCATGTTCGCCTCCTTGC | Os03g0316200 |
| *OsSUS3* | qSUS3 | GGCACCTGTCGTCAAAGCTGTT | TAGTCTCACGCACTCGCTGAGC | Os07g0616800 |
| *OsTPS8* | qTPS8 | TTGCACCATGCGACGAGAAGC | GCTCTTTGGCCTGGCATGATCC | Os08g0445700 |
| *OsPPT3* | qPPT3 | GGCACGGTGTTCACCAACATGA | TCGCACTCCAGAACCCAATCCA | Os01g0172100 |
| *GT2* | qGt2 | TGAAGTGCCAGTTGTTGCCATA | TCCTCAACCTCACGCCTGTAT | Os01g0762500 |
| *GSE5* | **Referring to the supplementary Table 7** | | | |
| *OsSSI* |
| *OsFLO2* |
| *FLO4* |
| *OsFLO5* |
| *OsFLO6* |
| *OsAmy3A* |
| *OsAPL2* |
| *OsRab5a* |
| *G6PIb* |

**Supplementary table 9 | Molecular marker genotype of some recombinant individuals during fine mapping of *qDEC5*.**

| Name of primers | | RM18004 | CH5-2 | RM18035 | CH5-37 | CH5-8 |
| --- | --- | --- | --- | --- | --- | --- |
| Position on chrom.5（bp） | | 4973068 | 5166337 | 5305663 | 5366715 | 5379417 |
| Lines with high PGWC and DEC | 23-7 | **H** | S | S | S | S |
|  | 26-8 | **H** | S | S | S | S |
|  | 22-6 | **H** | S | S | S | S |
|  | 11-2 | **H** | **H** | S | S | S |
|  | 15-7 | **H** | **H** | S | S | S |
|  | 3-8 | **H** | **H** | S | S | S |
|  | 5-1 | **H** | **H** | S | S | S |
|  | 27-8 | **H** | **H** | S | S | S |
|  | 1-2 | **H** | **H** | **H** | S | S |
|  | 7-7 | S | S | S | **H** | **H** |
|  | 10-7 | S | S | S | **H** | **H** |
|  | 13-1 | S | S | S | S | **H** |
|  | 27-1 | S | S | S | S | **H** |

H, Heterozygous genotype; S, the genotype of recurrent parent.

**Supplementary table 10 | Genes in the target interval.**

| Gene MSU ID | Start position | End position | Gene function |
| --- | --- | --- | --- |
| LOC_Os05g09460 | 5309681 | 5311945 | hypothetical protein |
| LOC_Os05g09470 | 5319265 | 5321928 | hypothetical protein |
| LOC_Os05g09480 | 5329581 | 5331490 | OsIAA16 - Auxin-responsive Aux/IAA gene family member, expressed |
| LOC_Os05g09490 | 5333726 | 5336905 | peptidase, T1 family, putative, expressed |
| LOC_Os05g09500 | 5337195 | 5341210 | hexokinase, putative, expressed |
| LOC_Os05g09510 | 5351970 | 5353052 | hypothetical protein |
| LOC_Os05g09520 | 5360431 | 5366701 | IQ calmodulin-binding motif family protein, expressed，*qSW5/GW5/GSE5* |

**Supplementary table 11 | Phenotyping of appearance qualities of rice cultivars with different haplotypes.**

| Haplotype | Number of lines | Traits | Average | Range |
| --- | --- | --- | --- | --- |
| *GSE5^ZJB^* | 39 | GL (mm) | 6.7±0.71 | 5.3-8.0 |
|  |  | GW（mm） | 2.1±0.25 | 1.6-2.7 |
|  |  | PGWC（%） | 36.1±30.5 | 0-96.5 |
|  |  | DEC（%） | 12.3±13.2 | 0-51.3 |
| *GSE5^ZB^* | 17 | GL (mm) | 5.7±0.34 | 5.2-6.7 |
|  |  | GW（mm） | 2.6±0.22 | 2.0-3.1 |
|  |  | PGWC（%） | 90.2±15.2 | 61.5-100.0 |
|  |  | DEC（%） | 45.2±20.6 | 15.0-79.8 |
| *GSE5^NIP^* | 13 | GL (mm) | 5.0±0.46 | 4.2-6.3 |
|  |  | GW（mm） | 2.7±0.16 | 2.4-2.9 |
|  |  | PGWC（%） | 20.8±17.3 | 3.0-63.5 |
|  |  | DEC（%） | 7.7±6.8 | 0.6-20.2 |

**Supplementary table 12 | Phenotyping of rice appearance qualities of three genotypes in BC_6_S_2_.**

| Group | Genotype | Items | GL（mm) | GW（mm) | L/W | PGWC（%） | DEC（%） |
| --- | --- | --- | --- | --- | --- | --- | --- |
| NIL*^qdec5^* | aa | Mean | 5.46 | 2.50 | 2.18 | 49.48 | 7.76 |
|  |  | SE | 0.01 | 0.01 | 0.01 | 2.13 | 0.39 |
|  |  | Min. | 5.3 | 2.3 | 2.0 | 0.0 | 0.0 |
|  |  | Max. | 5.6 | 2.6 | 2.4 | 82.0 | 15.9 |
|  |  | Number | 89 | 89 | 89 | 89 | 89 |
| NIL*^qDEC5^* | AA | Mean | 5.51 | 2.33 | 2.36 | 9.07 | 1.23 |
|  |  | SE | 0.01 | 0.01 | 0.01 | 1.58 | 0.24 |
|  |  | Min. | 5.2 | 2.2 | 2.1 | 0.0 | 0.0 |
|  |  | Max. | 5.7 | 2.6 | 2.5 | 61.0 | 9.8 |
|  |  | Number | 76 | 76 | 76 | 76 | 76 |
| NIL^+/-^ | Aa | Mean | 5.51 | 2.42 | 2.28 | 19.97 | 2.61 |
|  |  | SE | 0.01 | 0.00 | 0.01 | 1.04 | 0.16 |
|  |  | Min. | 5.3 | 2.3 | 2.1 | 2.0 | 0.2 |
|  |  | Max. | 5.7 | 2.6 | 2.4 | 74.0 | 13.1 |
|  |  | Number | 151 | 151 | 151 | 151 | 151 |

aa, Ressesive genotype of *GSE5*; Aa, Heterozygous genotype; AA, Dominance genotype.

**Supplementary table 13 | T-test for differences in the mean values of rice appearance qualities between aa or AA and Aa genotypes.**

| Genotype | *P*-Value | | | | |
| --- | --- | --- | --- | --- | --- |
|  | GL | GW | L/W | PGWC | DEC |
| aa & Aa | 0.0000 | 0.0000 | 0.0000 | 0.0000 | 0.0000 |
| AA & Aa | 0.4460 | 0.0000 | 0.0000 | 0.0000 | 0.0000 |

aa, Ressesive genotype of *GSE5*; Aa, Heterozygous genotype; AA, Dominance genotype.
